# Phenological plasticity enables thermal homeostasis in a wild bird population

**DOI:** 10.1126/sciadv.aeg5926

**Published:** 2026-04-24

**Authors:** David López-Idiáquez, Ella F. Cole, Charlotte E. Regan, Ben C. Sheldon

**Affiliations:** ^1^ Edward Grey Institute of Field Ornithology, Department of Biology, University of Oxford, Oxford OX1 3EL, UK.; ^2^ UK Centre for Ecology & Hydrology, Bush Estate, Edinburgh EH26 0QB, UK.

## Abstract

Shifts in the timing of seasonal events are widely documented biological responses to climate change, but measuring responses on human calendars reveals little about the underlying biological causes of such changes. Here, using six decades of individual-based data from wild great tits *Parus major*, we show that plasticity in reproductive phenology has enabled stable long-term thermal homeostasis despite marked local warming. This homeostasis has matched average temperatures at which reproductive success is maximized, enabling synchronization with a key invertebrate food source. Shifting our perspective from analyzing the phenological timing of life history events to analyzing changes relative to environmental gradients has the potential to shed light on the causes, mechanisms, and consequences of these responses by establishing direct links with biologically relevant variables.

## Teaser

Changes in phenology buffer against climate change by ensuring breeding at temperatures that maximize reproductive success.

## INTRODUCTION

Rapid climate change is altering environments across the globe, and, to persist, populations must respond to these changes. Long-term research on animal and plant populations has uncovered plastic and genetic responses to climate change in traits such as morphology (*1*), ornamentation (*2*), and phenology (*3*). These phenotypic changes, such as the general reduction in body size across taxa (*4*), have presumably helped populations to persist, despite the effects of climate change (*5*). Changes in reproductive timing are one of the best-described consequences of climate change (*6*), and the advance in egg-laying date of insectivorous birds to track the peak of food during breeding serves as a textbook example of phenological responses to global warming (*7*–*10*). Besides phenotypic changes in situ, an alternative ecological response to climate change is to track the optimal climatic niche through shifts in species and population distribution ranges. In general, as temperatures have risen, there is good evidence that species have moved their distributions poleward, to higher altitudes, or to greater depths, in search of cooler environments (*11*–*14*).

These two types of response could be viewed as equivalent for some populations, with changes in phenology (e.g., earlier timing) enabling populations to track optimal temperatures in situ (i.e., phenological thermal tracking hypothesis), allowing them to maintain a stable thermal niche without the need of a shift in their distribution ranges. This idea has been supported by three studies, one of plants (*15*) and two of birds (*16*, *17*), which were conducted at large spatial scales and across species and show that phenological changes can enable species to maintain stable climatic niches. For instance, in birds, it is suggested that temperature is better tracked through phenological changes than by shifts in altitude or latitude (*17*) and that, by advancing reproductive timing, Californian bird communities have buffered the impact of ~1°C increase in the area (*16*). Such population responses can, in principle, be produced by multiple mechanisms (e.g., evolution, plasticity, or redistribution of individuals), which cannot be disentangled using cross-sectional data collected across long time intervals. Individually-based longitudinal studies enable more direct dissection of the mechanisms that underlie such responses (*18*), including the direct assessment of the fitness consequences of varying temperatures at specific reproductive stages, but we are unaware of any such studies of the phenological thermal tracking hypothesis.

In this study, we explore the role of phenological change as a mechanism to track thermal niches during breeding by analyzing an individually based dataset comprising more than 12,000 breeding attempts within a great tit (*Parus major*) population from 1965 to 2023. In this population, as in many others of this species (*19*), egg-laying date has advanced by more than 2 weeks since 1965 in response to warming, making it an ideal study system to tackle this question (*3*, *8*). We analyze temporal trends in temperature from two different perspectives. First, based on calendar dates, we use a fixed temporal interval to quantify the change in temperature experienced during reproduction over the course of the study period. Second, we measure long-term temperature changes in five distinct time intervals defined by individual reproductive timing; each of these represents a different stage of bird reproduction, with potentially different sensitivities to temperature (*20*–*22*). Identifying significant temporal trends in temperature in the fixed intervals, but not in the intervals relative to individual reproductive timing, would imply that the observed advance in laying date has enabled great tits to maintain stable temperatures during reproduction. We then expand these analyses, linking temperatures in the different reproductive stages with multiple measures of reproductive success to analyze whether phenological thermal tracking has allowed great tits to maintain optimal temperatures during reproduction. Last, we use 45 years of data on winter-moth, *Operophtera brumata*, phenology from the same study site to analyze the link between relative temperatures and resource availability, as a potential mechanistic link between relative temperature and reproductive success due to its relevance for great tit fitness during nestling rearing (*23*–*25*).

## RESULTS

### Long-term stability in thermal environment

**Fig. 1.**
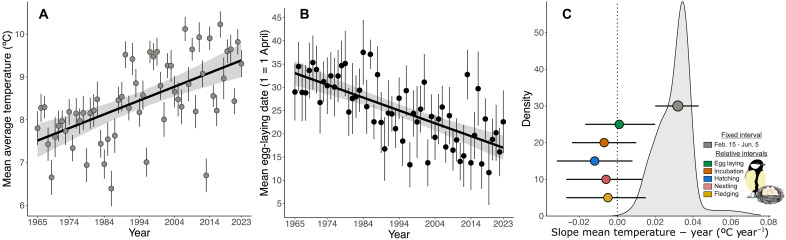
Temporal trends in temperature linked to great tit reproduction at Wytham woods near Oxford (UK). (**A**) Temporal trend in daily mean temperature [in degrees Celsius (°C)] in the interval between 15 February and 5 June between 1965 and 2023. Dots and whiskers represent the yearly mean temperature ± SE. (**B**) Temporal trend in first egg-laying date showing a 16.5-day advancement between 1965 and 2023; dots represent annual means ± SD. In (**C**), the gray dot represents the slope of the temporal trend in mean temperature between 15 February and 5 June, and the colored dots the slopes of the trends in mean temperature during empirically defined egg laying; incubation; and the hatching, nestling, and fledging periods. The whiskers represent the ±95% confidence intervals. The 95% confidence intervals crossing the dotted line at zero denote nonsignificant associations and hence temporal stability. The density plot is the distribution of the slopes computed for all intervals of between 8 and 15 consecutive days between 15 February and 5 June from 1965 to 2023.

Local mean temperature increased by 1.88°C across the fixed interval from 15 February to 5 June, corresponding to the reproductive period of great tits (see Materials and Methods), between 1965 and 2023 (0.032° ± 0.005°C year^−1^, *F*_1, 57_ = 31.21, *P* < 0.001, *n* = 59; Fig. 1A). As expected and extending previous data (*3*), mean first egg-laying date advanced by 16.5 days over the same period (−0.280 ± 0.039 days year^−1^, *F*_1,57.4_ = 50.77, *P* < 0.001; Fig. 1B). In contrast, when time intervals were defined on the basis of five components of individual reproductive timing, mean temperature remained very stable across all five periods between 1965 and 2023 (Fig. 1C and table S1). To explore potential biases introduced by comparing intervals of different lengths (the fixed interval is substantially longer than the relative intervals), we complemented our analysis of the fixed interval by exploring temporal trends in temperature for all periods of between 8 and 15 consecutive days (i.e., maximum and minimum duration of the relative intervals; see Materials and Methods) between 15 February and 5 June. The average slope estimate from these models is 0.030° ± 0.012°C year^−1^, closely aligning with the results obtained when using the full interval, and the distribution is very different from the relative slope estimates (Fig. 1C). Hence, the different patterns found in the fixed and relative approaches are not an artefact of the different interval lengths. Having established the stability of relative temperature, we then analyzed how temperature variation influenced individual fitness metrics.

### Links between the thermal environment and reproductive success

We analyzed the effects of mean temperature during the five relative reproductive stages on (i) the number of eggs laid (i.e., clutch size), (ii) the number of young fledged, (iii) the proportion of eggs laid that successfully fledged (i.e., fledging success), and (iv) the number of offspring recruited to the population (i.e., the number of recruits). These four measures provide a complementary and integrated perspective on reproductive success, related both to the number of genes that contribute to the next generation and the efficiency in achieving this contribution (i.e., investment required to produce a particular number of nestlings), in the short and long terms.

**Fig. 2.**
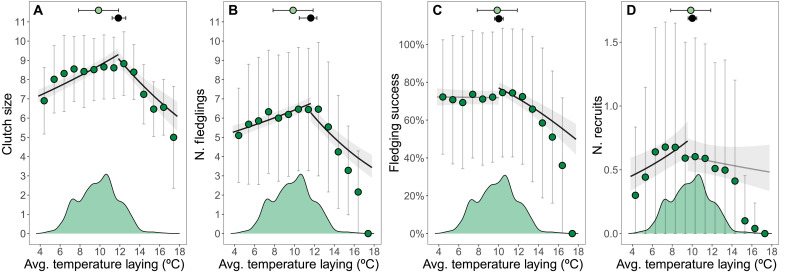
Associations between mean temperature at egg laying and reproductive success in great tits between 1965 and 2023. Lines and ribbons represent the predicted association and 95% credible intervals from the models. Black and gray lines, respectively, represent associations supported (95% credible intervals not including zero) and not supported (95% credible intervals including zero) by the models. Green dots and vertical whiskers represent (**A**) the raw average clutch size, (**B**) the number (N.) of fledglings, (**C**) fledging success, and (**D**) the number (N.) of recruits ±1 SD grouped in 1°C bins. Note that the sample size within each bin varies. Density plots represent the distribution of temperatures at egg laying (and hence give information about the sample size). The black dot and horizontal whiskers represent the temperature at which reproductive success is maximized and its 95% CIs. The green dot and horizontal whiskers represent the mean temperature of the period ± 1 SD.

For clutch size (analyzed only in relation to temperature at egg laying as the other periods occur after clutch completion), the model showed a strong hump-shaped association (Fig. 2A and table S2). Clutch size decreased with lower and higher temperatures than 11.82°C ([11.23, 12.58]; prepeak trend, 0.053 [0.042, 0.064]; postpeak trend, −0.059 [−0.081, −0.038]).

The number of young fledged also showed a hump-shaped association with temperature at egg laying, decreasing with lower and higher temperatures than 11.61°C ([10.39, 12.19]; prepeak trend, 0.050 [0.037, 0.063]; postpeak trend, −0.087 [−0.113, −0.061]; Fig. 2B and table S3). During the hatching, nestling, and fledging periods, the number of young fledged slightly decreases at temperatures lower and higher than the peak. Last, the number of fledglings showed a weak positive association with temperature during incubation (tables S4 to S7 and figs. S1 to S4).

Analysis of fledging success also revealed a nonlinear association with temperature at egg laying, which was driven by a decrease in fledging success at temperatures higher than the peak (10.01°C [9.59, 10.46]; prepeak trend, −0.006 [−0.074, 0.061]; postpeak trend, −0.310 [−0.385, −0.234]; Fig. 2C and table S8). Fledging success also showed a nonlinear association with temperature in the other periods, but, in all cases, the trends after and before the peak were weak and not always supported by the model (tables S9 to S12 and figs. S1 to S4).

For the number of recruits, we found decreases at temperatures during egg laying below 9.60°C [7.95, 11.60], with nonsupported changes after the peak (prepeak trend, 0.093 [0.037, 0.148]; postpeak trend, −0.026 [−0.082, 0.030]; Fig. 2D and table S13). Links between the number of recruits and temperature in the other periods were mostly not supported by our models, with only a positive prepeak association with temperature during the hatching period (tables S14 to S17 and figs. S1 to S4).

### Phenological thermal tracking by winter moths

**Fig. 3.**
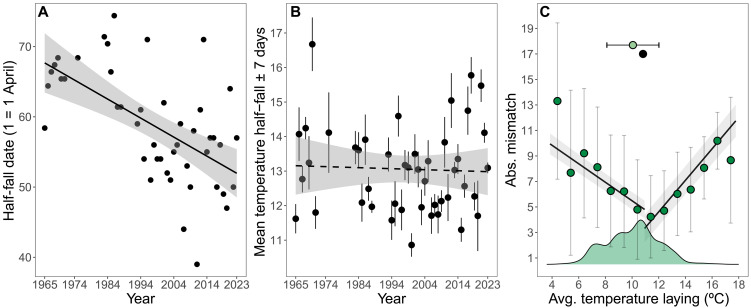
Phenological thermal tracking by winter moths and synchrony with great tit phenology. Temporal trends in (**A**) the half-fall date (i.e., the date by which 50% of the seasonal total caterpillars descending from trees to pupate are caught in water traps) and (**B**) in average temperature [in degrees Celsius (°C)] ± SE at the half-fall date ± 7 days in winter moths at Wytham Woods. (**C**) Relationship between temperature at laying and absolute (Abs.) mismatch. Lines and ribbons represent the predicted association and 95% credible intervals from the models. The green dots and whiskers represent the average predicted mismatch ± 1 SD. Density plots represent the distribution of temperatures at great tit egg-laying (and hence give information about the sample size). The black dot and horizontal whiskers represent the temperature at which mismatch is minimized and its 95% credible interval. The green dot and horizontal whiskers represent the mean temperature of the period ± 1 SD.

A key food resource for nestling great tits is lepidopteran larvae, which can comprise more than 80% of their diet (*26*–*28*). Long-term studies of winter moth caterpillars have shown that the timing of their peak abundance is linked to annual climate variation and, for passerine birds like the great tit, that synchrony with peaks of this frequently abundant species is associated with higher reproductive output (*23*–*25*, *29*, *30*). Further, winter moth peak abundance is temporally correlated with that of other phytophagous insects (*31*, *32*). Thus, understanding temporal changes in winter moth abundance may provide a mechanistic explanation for associations between great tit reproductive success and temperature. Analysis of a standard measure of the timing of winter moth larval biomass (half-fall date, i.e., the date by which 50% of the seasonal total final-instar caterpillars descending from trees to pupate are caught in water traps; see Materials and Methods), from 45 years that were available between 1965 and 2023, showed that the seasonal phenology of winter moths has advanced by ~16 days since 1965 (−0.270 ± 0.056 days year^−1^, *F*_1,43 _= 23.26, *P* < 0.001, *n* = 45; Fig. 3A). As was the case for great tit phenology (Fig. 1C), temperature in the period around winter moth half-fall (±7 days) has not changed between 1965 and 2023 (−0.003° ± 0.011°C year^−1^, *F*_1,43_ = 0.119, *P* = 0.731, *n* = 45; Fig. 3B).

### Links between thermal niche and resource overlap

**Fig. 4.**
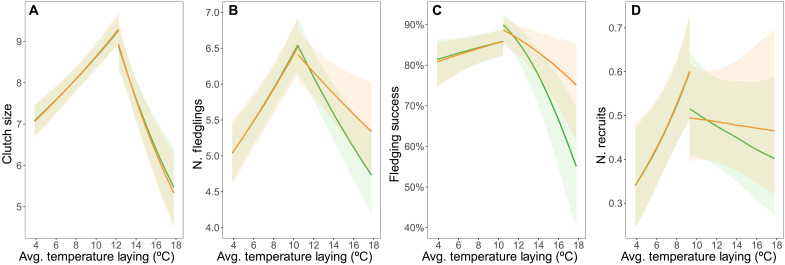
Associations between temperature in the egg-laying period and reproductive success of great tits weaken when controlling for mismatch with winter moth caterpillar timing. Green lines and ribbons represent the predicted association and 95% confidence interval of the models not including mismatch as a covariate in the models. Orange lines and ribbons represent the predicted associations when accounting for mismatch. Note that the sample sizes in these models differ from those in Fig. 2 due to reduced information on winter moth annual half-fall dates (see Materials and Methods).

Using (i) the individual-level data on breeding dates of great tits between 1965 and 2023 and (ii) annual data on standard measures of winter moth timing, we calculated a measure of matching between timing of tit breeding and resource availability as follows: match = (hatching date + 10) − half-fall date. The rationale for this measure is that energetic demand of nestlings is maximized at around 10 days of age (*24*) and that late instar larvae provide the highest nutritional value. We then asked how individual-level temperature during specific reproductive periods related to the absolute value of the predicted match with the resource peak. We found nonlinear associations between temperature in all four reproductive periods (the fledging period was not included in this analysis as it frequently occurs after the caterpillar half-fall date) and absolute mismatch with winter moth caterpillar peak (table S18). Breeding at warmer and colder temperatures than local optima increased the mismatch with the peak of caterpillar availability during egg-laying (Fig. 3C and table S19), incubation, and the hatching and nestling periods (see fig. S5 and table S19). Hence, these analyses are consistent with the suggestion that matching timing with a key trophic level is an important factor in driving the thermal homeostasis across specific reproductive stages in great tits. A role for mismatch with caterpillar availability in mediating the link between temperature and great tit reproductive success is supported by the fact that for the two most closely linked reproductive success metrics (number of fledglings and fledging success), the associations between reproductive success and temperature weaken markedly when controlling for mismatch (Fig. 4 and tables S20 and S21; see Materials and Methods), although additional mechanisms, perhaps involving direct effects of temperature and other trophic interactions, must also operate.

## DISCUSSION

Using an individually-based dataset comprising more than 12,000 breeding attempts, we demonstrate that the 16.5-day phenological advancement in timing of breeding over six decades in our population has allowed great tits to maintain stable temperatures during reproduction, buffering the almost 2°C increase in average temperature observed between 1965 and 2023*.* This finding supports the untested hypothesis that phenological adjustments enable populations to track their thermal niches without the need of geographical range shifts. Through the tracking, great tits have bred at temperatures that maximize reproductive success. Overall, our results suggest that the stasis in temperature during breeding is consistent with natural selection acting to maintain the population at a local optimum.

Phenological changes are a well-described response to climate change, with much existing research focusing on changes in reproductive timing measured relative to human calendar dates (*33*, *34*). However, the environmental consequences of changes in phenology have received less attention (*15*–*17*). Here, we show that the phenological advancement of our population has resulted in what is effectively thermal homeostasis during great tit reproduction, despite rising temperatures over six decades (Fig. 1). This confirms the previous results from two studies at the community level (*16*, *17*), but using individual-based phenotypic and fitness data at considerably higher spatial and temporal resolution. Our results also align with recent studies focusing on optimal reproductive timing (*10*, *35*). de Villemereuil *et al*. (*35*) found that several mammal and bird populations exhibit fluctuations in optimal reproductive timing, with relatively early or late breeding being favored in different years. Our analyses suggest that these fluctuations might represent an individual adjustment in reproductive timing as a response to the environmental change to breed at optimal temperatures or, in other words, that the changes in optimal reproductive timing are a consequence of tracking their thermal niche during breeding. Youngflesh *et al*. (*10*), analyzing banding data from 41 bird species across North America, found that breeding productivity decreased when breeding deviated from a phenological optimum. Our analyses linking temperatures during breeding to reproductive success add an individual-level analysis and mechanistic aspect to this general pattern. We found that several measures of reproductive success in great tits at Wytham are maximized at intermediate breeding temperatures. This was particularly true for temperatures at egg laying, which showed the strongest associations with reproductive success, probably because egg laying is the period during which great tits have the greatest scope to adjust their reproductive investment. Thus, our results are consistent with natural selection having acted to enable tracking local optimal temperatures during breeding, especially during egg laying, as is highlighted by the close alignment of average temperatures in this period with those that maximize reproductive success in our population (Fig. 2).

Associations between temperature in the relative reproductive periods and reproductive success might, in principle, be explained by the direct effects of temperature on adults and nestlings (*16*, *36*). Temperature experienced by the embryo during incubation and by nestlings and fledglings can directly impact metabolic and growth rates and also fledging success (*37*, *38*) and survival (*39*, *40*). A complementary and nonexclusive explanation is that indirect effects of temperature on reproductive success alter synchrony with resource availability (*41*). While tits can maintain some degree of synchronization through changes in hatching date (*42*, *43*), the different effects of thermal environments on development rates of endotherms and exotherms are expected to lead to mismatch if temperatures are unusually cool or warm (*7*). This could lead to greater mismatch with the peak of food demand by nestlings, thus increasing the relative costs of reproduction for adults (*44*). Our results support indirect effects of temperature on reproductive success as at least part of the mechanism, as we identified optimum local temperatures in relative breeding periods that minimize the mismatch between the peak of food availability and demand. High and low temperatures are expected to, respectively, accelerate and retard caterpillar development; thus, it is plausible that the increased mismatch occurs because of a limited capability of great tits to adjust the developmental rate of offspring after the clutch has been laid. Besides, great tit ability to adjust the timing of hatching is greater in cooler than warmer conditions (*43*). This supports the stronger association between mismatch and temperature at egg laying after than before the peak (table S19), which also aligns with the stronger overall fitness penalties of breeding at higher than lower temperatures (table S22).

The proximate mechanisms by which phenological thermal tracking is achieved are not known. Research on reproductive timing of woodland passerines in a climate change context has explored two main cues used to time the start of reproduction, temperature, and tree phenology. Experimental work has shown that captive great tits directly respond to changes in average temperature and warming rates (*45*, *46*), although responses differ from those of wild birds exposed to the same temperature profiles, suggesting the operation of other cues. Tree phenology, in this case, bud-burst dates, is an additional cue that might be used by great tits to time the start of breeding [(*46*–*48*), but see (*49*–*51*)]. In our population, both temperature (*8*) and tree phenology (*31*, *49*) have been identified as relevant factors modulating great tit reproductive timing. Thus, it is possible that the direct effects of temperature and indirect effects of phenology at other trophic levels allow great tits to track their thermal niche during breeding. Uncertainty about the proximate mechanisms to track thermal niches through phenology is not exclusive to woodland birds. Across taxa, from amphibians to mammals and insects, the relative contributions of temperature, resource availability, or photoperiod sensitivity as phenological cues remain to be clarified. Disentangling the role of these factors and others in free-ranging populations will be a challenging task.

Phenological thermal niche tracking of the kind demonstrated here provides an assessment of the extent to which populations are responding to climate change assessed against a more biologically relevant measure than calendar date. In our study, long-term data on caterpillar half-fall dates showed that the timing of this event has advanced by ~16 days between 1965 and 2023. Winter moth caterpillars face strong selection to synchronize their emergence to the availability of newly emerged leaves, as mismatch with tree budburst imposes strong fitness costs both in terms of survival and weight at pupation (*52*). In this context, it would be expected that, thanks to their phenological advancement, winter moths have been able to maintain stable temperatures as caterpillars, although we lack detailed long-term phenological information about life stages other than the fifth instar reported here. Hence, these data suggest that phenological thermal tracking may also occur beyond the tri-trophic (birds, insects, and trees) system that we study, with special relevance in systems relying on ephemeral resources during reproduction.

Overall, our analyses show that the behavioral responses to climate warming in our study population have allowed great tits to maintain stable temperature during breeding. Viewing individual birds’ decisions about when to breed as a choice of a position on relevant environmental gradients (e.g., temperature or rainfall), rather than the choice of a date, offers the opportunity for a fresh perspective on the causes, mechanisms, and consequences of phenological responses to climate change. Scaling this perspective up to different populations and different species will reveal the generality of the pattern described here. Scaling it down by using environmental data collected at smaller scales (e.g., at scales relevant to individuals) has the potential to reveal a previously unexplored understanding of the mechanisms behind phenotypic plasticity to changing environments. This perspective, of measuring responses against biologically relevant gradients, could also be extended to the analysis of the effects of other forms of environmental change.

## MATERIALS AND METHODS

### Study system

We carried out this study in a nest box population of great tits at Wytham Woods (51°46′N, 1°20′W), a mixed deciduous woodland in Oxfordshire, which has been continuously monitored since 1947 (*53*). Population monitoring includes nest box visits at least once a week during the breeding season (April to June), enabling the collection of detailed breeding data including laying date (date in which the first egg is laid), clutch size, hatching date, and fledgling success for each pair. Data on winter moth half-fall dates at Wytham were supplied by L. Cole. The half-fall date is the day on which 50% of the seasonal total of winter moth caterpillars are collected in water traps under oak trees and provides a robust index of the peak of food availability for great tits (*3*, *31*). In this study, as in previous work (*20*), we define the starting year in 1965 to avoid potential biases introduced by the provision of new boxes up to 1961; mean generation time of a great tit is <2 years (*54*). Winter moth half-fall data were available for 45 of those 59 years (nonavailable years: 1972–1974, 1976–1982, and 1989–1992; analysis of great tit data remains qualitatively the same when using this reduced dataset; see tables S23 to S40).

### Temperature data and period definition

We obtained daily mean temperature data from the Met Office Hadley Centre. In particular, we used the Central England Temperature dataset (www.metoffice.gov.uk/hadobs/hadcet/data/download.html). To analyze temporal trends in temperature between 1965 and 2023, we followed two approaches. First, we used a fixed interval spanning 15 February to 5 June. The beginning of the interval that best explains the onset of egg laying in our population is 15 February, which was obtained using a sliding window analysis (see supplementary text 1 for further information). To capture temperature across the whole breeding season, we expanded the interval until 5 June, which represents the average end of breeding in our population, computed as the addition of average laying date, average incubation duration, and 20 days (i.e., approximate time between hatching and fledgling). Second, we examined temperature changes in five periods relative to the observed timing of each individual reproductive event (*20*–*22*). This included egg laying (period from the first to the last egg); incubation (from the start of incubation to hatching); and the hatching (from hatching to 7 days posthatch), nestling (from 8 to 15 days posthatch), and fledgling (from 16 to 21 days posthatch) periods. We did this by matching daily meteorological data with each individual breeding attempt (see fig. S6). For instance, temperature prevailing at each individual breeding attempt during the egg-laying period was computed as the average temperature in the days between laying of the first and last eggs. These periods represent different stages in great tit breeding and, although they have a Markovian nature, likely show different sensitivities and responses to temperature changes in them. For instance, while the start of egg laying has the largest effect on phenology, great tits can fine-tune their timing through incubation maximizing the match with the peak of food availability. This cannot happen during the subsequent reproductive stages. In those, temperature may have direct and indirect effects on the brood, as the hatching period captures the stage when nestlings are only starting to thermoregulate and the nestling period the stage when food requirements are highest.

### Statistical analyses

#### Temporal trends in laying date

To analyze the temporal trends in egg-laying date, we fitted a linear mixed effect model (LMM) that included April egg-laying dates as a dependent variable and year (as a continuous variable) as explanatory. To control for the nonindependence of data taken on the same years and from the same individuals, we included year (as a categorical variable) and female identity as random effects. See supplementary text 2 for more information on the results from this model.

#### Temporal trends in temperature using fixed and relative intervals

To analyze the temporal trends in mean temperature on the fixed interval (from 15 February to 5 June), we fitted a linear model that included the yearly average temperature in that period as a dependent variable and year (as a continuous variable) as fixed effect. To investigate the potential bias introduced by the selected length of this interval (e.g., longer intervals may be less prone to stochastic variation), we repeated this analysis with the same model structure for all intervals of between 8 and 15 consecutive days, which represent the minimum and maximum length of the relative intervals and, thus, a more biologically relevant and comparable time frame than the whole interval. For an alternative approach to analyze the phenological thermal niche tracking hypothesis, see supplementary text 3.

To analyze the temporal trends in temperature in the relative periods, we fitted one LMM per period. In these models, mean temperature in each individual breeding attempt and period was included as a dependent variable and year (as a continuous variable) as a fixed effect in all models. To control for the nonindependence of data taken on the same years and from the same individuals, we included year (as a categorical variable) and female identity as random effects. Analyzing temperature changes from a within-individual perspective yields similar results; for further information, see supplementary text 4.

#### Association between temperature during the relative intervals and reproductive success

We analyzed the association between temperature in the relative periods and four proxies of breeding success: clutch size, number of fledglings, fledgling success and number of recruits, in two steps. First, we used generalized LMMs (GLMMs) with natural cubic splines with seven degrees of freedom to identify the temperature values that maximized breeding success in a Bayesian framework. Then, we used these temperature values to analyze the pre- and postpeak trends using regular GLMMs (using different degrees of freedom did not changed substantially the temperature peaks found by the models; see supplementary text 5).

The models of clutch size assumed a Poisson error distribution and included mean temperature of the period with a natural cubic spline in addition to laying date and number of neighbors as fixed effects. Number of fledglings and recruits was modeled with the same structure as clutch size but assuming a zero inflated Poisson error distribution. Number of recruits was computed as the number of offspring breeding in Wytham as adults in the successive breeding seasons. We included breeding data from 2024 and 2025 to improve the accuracy of our number of recruit estimates. We modeled fledging success assuming a binomial distribution of the number of successes (number of fledglings) out of the number of trials (clutch size). These models included mean temperature of the period with a natural cubic spline as fixed effects in addition to laying date, clutch size, and number of neighbors. Accounting for any between year effects yields similar results to those obtained from these models, showing that the patterns found are not a byproduct of interannual differences in fitness and temperature (see supplementary text 6 for further information). From these models, we obtained the temperatures that maximized each reproductive trait. Using those values, we analyzed the associations between temperature and reproductive success at both sides of the peak. These models had the same structure as those previously described, apart from temperature, which was included as a linear term.

To explore the effects of mismatch on the strength of the associations between reproductive success and temperature in the egg-laying period, we fitted models with the same structure than above but also including absolute mismatch as a covariate. All models in this section included year (as a categorical variable) and female identity as random effects, and all fixed effects were scaled to a mean of 0 and an SD of 1.

#### Temporal trends in half-fall dates and phenological thermal tracking in winter moths

We analyzed the temporal trends in half-fall dates by fitting a linear model with the half-fall date (as the number of days since 1 April) as a dependent variable and year (as a continuous variable) as fixed effect. To analyze the temporal trends in temperature at half-fall date, we computed the yearly average temperature ± 7 days around half-fall and included this information as a dependent variable in a linear model with year (as a continuous variable) as fixed effect. To explore how the selection of the intervals length influenced our results, we repeated this analysis using ±15- and 20-day intervals, with consistent results in all of them (table S41).

#### Temperature during the relative periods and mismatch with peak of caterpillar availability

We computed the mismatch between the peak of food demand and the peak of food availability as the absolute number of days between the date when nestlings are 10 days old (i.e., peak of food demand) and half-fall date (*7*). To analyze the link between temperatures at the relative periods and mismatch, we fitted one LMM per period that included absolute mismatch as a dependent variable and the temperature of the period with a spline with seven degrees of freedom as explanatory in brms assuming a Gaussian distribution of errors. In these models, we included year (as a categorical variable) and female identity as random effects. In this set of analyses, we did not include the fledging period as it occurs mostly after the peak of food availability. Note that the sample size differences in this set of models are caused by missing half-fall data in some years. The degree of mismatch between the peak of food demand and the peak of caterpillar availability has not changed across the study period (see supplementary text 7)

All models were fitted using R [v. 4.3.1; (*55*)] and the packages lme4 [v. 1.1-35.5; (*56*)] and brms (*57*) to fit the linear models, LMMs and GLMMs, and splines (v. 4.4.1) to fit the splines. In the linear models we visually inspected the distribution of model residuals, not finding strong evidence for deviations from normality. Bayesian models were run for 10,000 iterations across four chains with a warm-up of 1000 iterations and a thin of 10. For each analysis, we assessed the convergence using trace plots by assessing effective sample sizes and using the Gelman-Rubin convergence diagnostic. The predicted trends shown in the plots were obtained using the package ggeffects [v. 1.3.1; (*58*)], and the 95% confidence interval was obtained using the package confintr [v. 1.0.2; (*59*)].

## Supplementary Materials

### This PDF file includes:

Supplementary Text Figs. S1 to S11 Tables S1 to S46 References
