# Supplementary material for: Phenological plasticity enables thermal homeostasis in a wild bird population
Source: Sci Adv. 2026 Apr 24;12(17):eaeg5926. doi: 10.1126/sciadv.aeg5926 (PMC13108551; doi:10.1126/sciadv.aeg5926)
Supplement: Supplementary file 1 — Supplementary Text Figs. S1 to S11 Tables S1 to S46 References [file sciadv.aeg5926_sm.pdf]

Science Advances
AAAS

Supplementary Materials for

Phenological plasticity enables thermal homeostasis in a wild bird population

David López-Idiáquez *et al.*

Corresponding author: David López-Idiáquez, david.lopez-idiaquez@biology.ox.ac.uk

Sci. Adv. **12**, eaeg5926 (2026)
DOI: 10.1126/sciadv.aeg5926

This PDF file includes:

Supplementary Text
Figs. S1 to S11
Tables S1 to S46
References

Supplementary Text (ST)

ST1: We used the R package *climwin* (v. 1.2.3; 60) to estimate the window where mean temperature was most strongly associated with the onset of reproduction in our population. We used the same model specifications as in Simmonds et al. (2019; 61) who successfully used this approach in a subset of our data. We used absolute sliding time windows, with the 20th of May as reference day. We allowed windows to vary 365 days in length. We found that the temperature window that best explained the onset of reproduction in our data started the 15th of February and ended the 2nd of May. We used the critical probability to check whether the window we detected was just by chance. With that aim, we ran 5 randomised models using different randomisations of the onset of reproduction. We used these randomisations and the original model to compute the critical probability which was p<0.001. Smaller p-values are associated with a smaller probability of the window we detected was by chance.

ST2: The results from the linear mixed effect model analysing the temporal trends in egg-laying dates show that laying date has advanced 16.5 days between 1965 and 2023 (-0.280±0.039, $F_{1,57.4}$=50.77, p<0.001).

ST3: As a complementary approach to test that isothermy has been achieved through phenological thermal tracking we analysed the counter-factual. That is, we asked how the temperature at which each breeding interval occurs would have changed in the absence of phenological change over time. To do so we computed the breeding intervals using the first ten years of the series, then computed the average temperature each year in that period and finally explored the temporal trends in temperature using those data. With that aim, and for each year in the first decade (1965-1974) we computed the start and end of each period of interest (egg-laying, incubation…) using the 0.025 and 0.975 quantiles and averaged them to get an overall start and end of our periods of interest. Then using those time intervals, we computed the average temperature experienced each year. Analysing the temporal trends in temperature in those intervals shows a consistent and significant increases in temperature (Table S42, Fig. S7), strongly suggesting that in the absence of a phenological change, great tits in our population would have experienced warmer temperatures during breeding.

ST4: We analysed whether the temperature stability in the five considered periods was also reflected at the within-individual level, that's it that temperature at which individuals breed is stable through their lives. We did that using a within-subject centring method (62). We mean-centred the temperatures experienced at each period within each female and regressed those mean-centred values against the breeding attempt each temperature was experienced. We did that by fitting separate linear mixed models for each period with the within-individual component of temperature as dependent variable and breeding attempt and age (juvenile vs adult) explanatory terms. Year was included as a random effect. To control for the heterogeneity introduced by individuals breeding a variable number of times, whose temperatures may be biased by stochastic between-year variation the models included a weight to give more relevance to those females breeding a higher number of times.

The results of these analyses show that temperature has changed very little at the within-individual level across the five considered periods (Table S43, Fig. S8). During egg-laying and the hatching

and fledgling periods temperature does not show any significant change. Temperature during incubation and the nestling period shows respectively a significant increase and decrease over the lifetime of an individual. These changes are, however, weak. Considering that the mean number of breeding attempts of those females that breed more than once is 2.6, the significant changes during incubation and the nestling period imply respectively a temperature change of ~ -0.049º C and 0.091º C.

The results from this complementary analysis further strengthen our conclusions, as they show either within-individual stability or very little change in temperature across the life of an individual, which especially true for the egg-laying period, the most relevant period according to our results.

ST5: To explore the influence of the selection of the degrees of freedom (df) of the splines in our generalised linear mixed models we re-ran the models with two alternative dfs enabling more (df=9) and less (df=5) flexibility to the trend. The temperatures that maximised reproductive success are consistent regardless of the method selected to obtain them (ST5-Table 1). Then we analysed the trends at each side of the peaks obtained with the three different approaches, finding overall consistent trends between methods (S44).

ST6: To explore whether our patterns linking temperature during egg-laying and reproductive success were biases the by between-year differences in reproductive success and/or temperature, we combined the results from the models shown in the main text with two alternative approaches that allowed us to control for the between year effects.

First, we ran models with the same structure as those in the main text but including a random intercept and slope for the quadratic component of temperature in a frequentist framework. We included temperature and its quadratic component in the random slope model, as the splines led to convergence issues. Despite being simpler, this approach does not affect our ability of exploring whether the patterns are driven by between year effects, and the temperatures that maximise reproductive success are similar using both methods. Second, we fitted Bayesian models with the same structure as those in the main text but using year centred values of reproductive success and not including year as random.

The results from these complementary approaches yield very similar optimal temperatures for the four reproductive success metrics we considered (Table S45). The only difference lays on the optimal temperature for number of recruits when using the year centred values, which despite being similar to those obtained with the other methods it was estimated with high uncertainty. The patterns drawn from these alternative approaches, the population level pattern when considering the random slope models, show similar trends to those shown in the main text (Fig. S9). Overall, we think that this suggest that the associations we show in the main text are not a byproduct of between year differences.

We followed a similar approach to analyse whether any between year pattern was driving our models linking temperatures during the breeding periods and mismatch with the peak of food availability. Temperatures that minimize mismatch obtained using the alternative analytical approaches and those by the models presented in the main text are consistent (Table S46),

especially during the egg-laying period (Fig. S10). This strongly suggests that our conclusions regarding the links between temperature at egg-laying and mismatch between the peak of food availability and demand are not biased by a between-year pattern.

ST7: We analysed the temporal trends in mismatch between the peak of food demand by the great tits and the peak of winter moth caterpillar availability by fitting a linear mixed effect model with normal distribution of errors. Mismatch was included as dependent variable and year as fixed effect. The model included year and mother as random effects.

The result from this model shows that mismatch has remained stable across the study period (0.007±0.037, $F_{1,43.56}$=0.038, p=0.846, n=9711; Fig. S11).

Figure S1. Associations between mean temperature during incubation and reproductive success. Lines and ribbons represent the predicted association and 95% credible intervals (CI) from the models at both sides of the peak. Black and grey lines respectively represent associations supported (95% CI not including zero) and not supported (95% CI including zero) by the models. Orange dots and vertical whiskers represent the raw number of fledglings (**A**), fledging success (**B**) and number of recruits (**C**) ± 1 standard deviation grouped in 1º C bins. Note that the number of samples within each bin varies. Density plots represent the distribution of temperatures during incubation. The black dot and horizontal whiskers represent the temperature at which reproductive success is maximized and its 95% CIs. The orange dot and horizontal whiskers the mean temperature of the period ± 1 standard deviation.

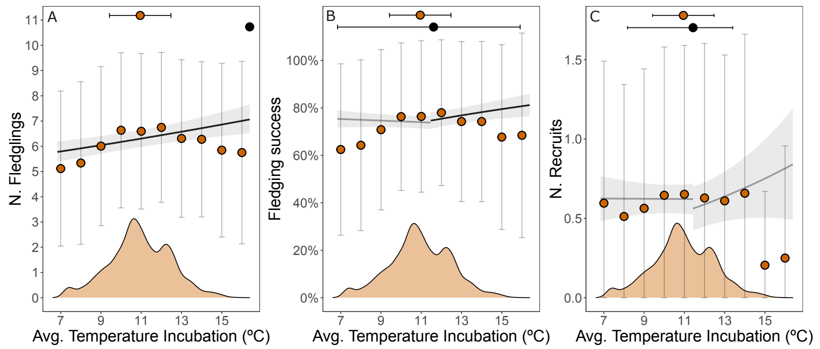

Figure S2. Associations between mean temperature during the hatching period and reproductive success. Lines and ribbons represent the predicted association and 95% credible intervals (CI) from the models at both sides of the peak. Black and grey lines respectively represent associations supported (95% CI not including zero) and not supported (95% CI including zero) by the models. Blue dots and vertical whiskers represent the raw number of fledglings (**A**), fledging success (**B**) and number of recruits (**C**) ± 1 standard deviation grouped in 1º C bins. Note that the number of samples within each bin varies. Density plots represent the distribution of temperatures during the hatching period. The black dot and horizontal whiskers represent the temperature at which reproductive success is maximized and its 95% CIs. The blue dot and horizontal whiskers the mean temperature of the period ± 1 standard deviation.

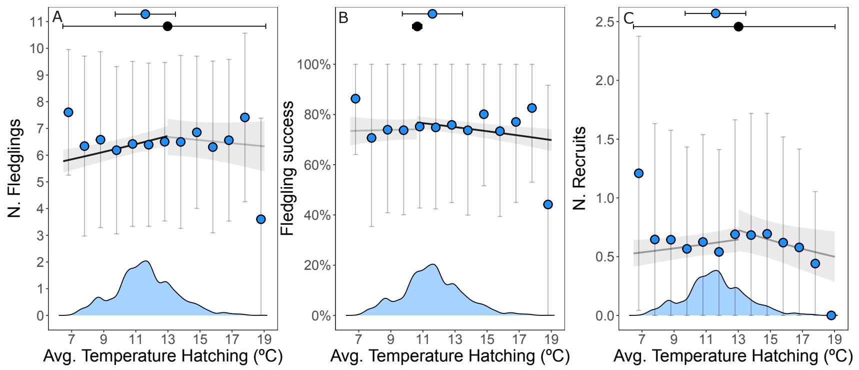

Figure S3. Associations between mean temperature during the nestling period and reproductive success. Lines and ribbons represent the predicted association and 95% credible intervals (CI) from the models at both sides of the peak. Black and grey lines respectively represent associations supported (95% CI not including zero) and not supported (95% CI including zero) by the models. Red dots and vertical whiskers represent the raw number of fledglings (**A**), fledging success (**B**) and number of recruits (**C**) ± 1 standard deviation grouped in 1º C bins. Please note that the number of samples within each bin varies. Density plots represent the distribution of temperatures during the hatching period. The black dot and horizontal whiskers represent the temperature at which reproductive success is maximized and its 95% CIs. The red dot and horizontal whiskers the mean temperature of the period ± 1 standard deviation.

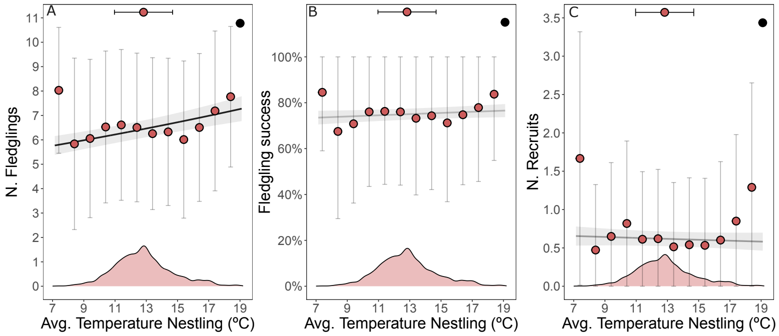

Figure S4. Associations between mean temperature during the fledging period and reproductive success. Lines and ribbons represent the predicted association and 95% credible intervals (CI) from the models at both sides of the peak. Black and grey lines respectively represent associations supported (95% CI not including zero) and not supported (95% CI including zero) by the models. Yellow dots and vertical whiskers represent the raw number of fledglings (**A**), fledging success (**B**) and number of recruits (**C**) ± 1 standard deviation grouped in 1º C bins. Please note that the number of samples within each bin varies. Density plots represent the distribution of temperatures during the hatching period. The black dot and horizontal whiskers represent the temperature at which reproductive success is maximized and its 95% CIs. The yellow dot and horizontal whiskers the mean temperature of the period ± 1 standard deviation.

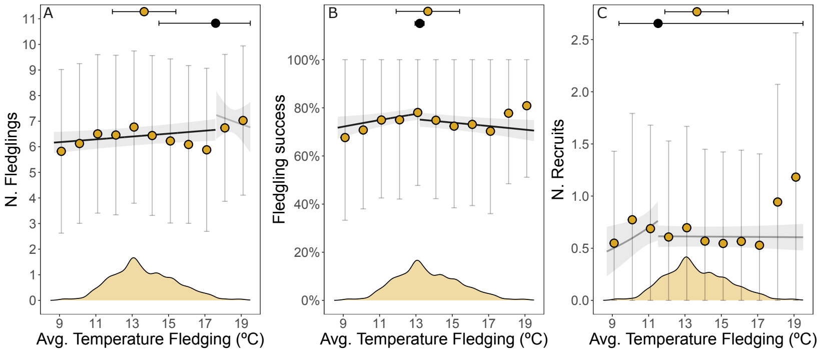

Fig. S5. Associations between mean temperature and mismatch. Lines and ribbons represent the predicted association and 95% credible intervals (CI) from the models. The orange, blue and red dots and whiskers represent the average predicted mismatch ± 1 standard deviation respectively for incubation (**A**), hatching (**B**) and nestling periods (**C**). Density plots represent the distribution of temperatures in each period relative to great tit breeding. The black dot and horizontal wishers represent the temperature at which mismatch is minimised and its 95% CI in each period. The coloured dots and horizontal wishers the mean temperature of the period ± 1 standard deviation.

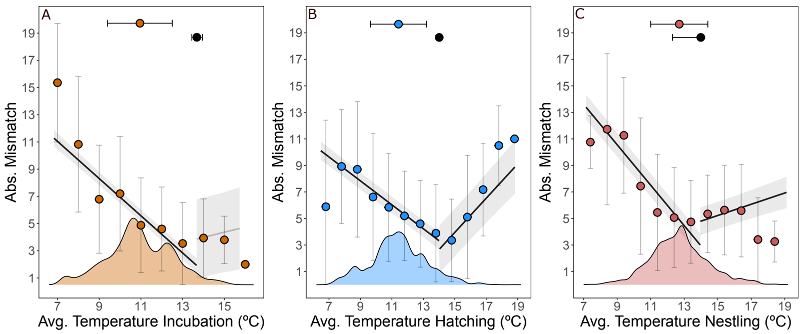

Figure S6: Illustrative figure of the used followed to compute average temperature during periods relative to the timing of each individual breeding attempt, using two broods in the year 1988 as an example. Julian date represents the number of days since April 1st, and Temp. (º C) represents the average temperature each day. Green represents the egg-laying period, the interval between laying the first and last egg. Orange represents the incubation period, the interval between laying the last egg and hatching. Blue represents the hatching period, the interval between hatching and 7-days post-hatch. Red represents the nestling period, the interval between 8- and 15-days post-hatch. Grey represents the fledging period, the interval between 16- and 23-days post-hatch.

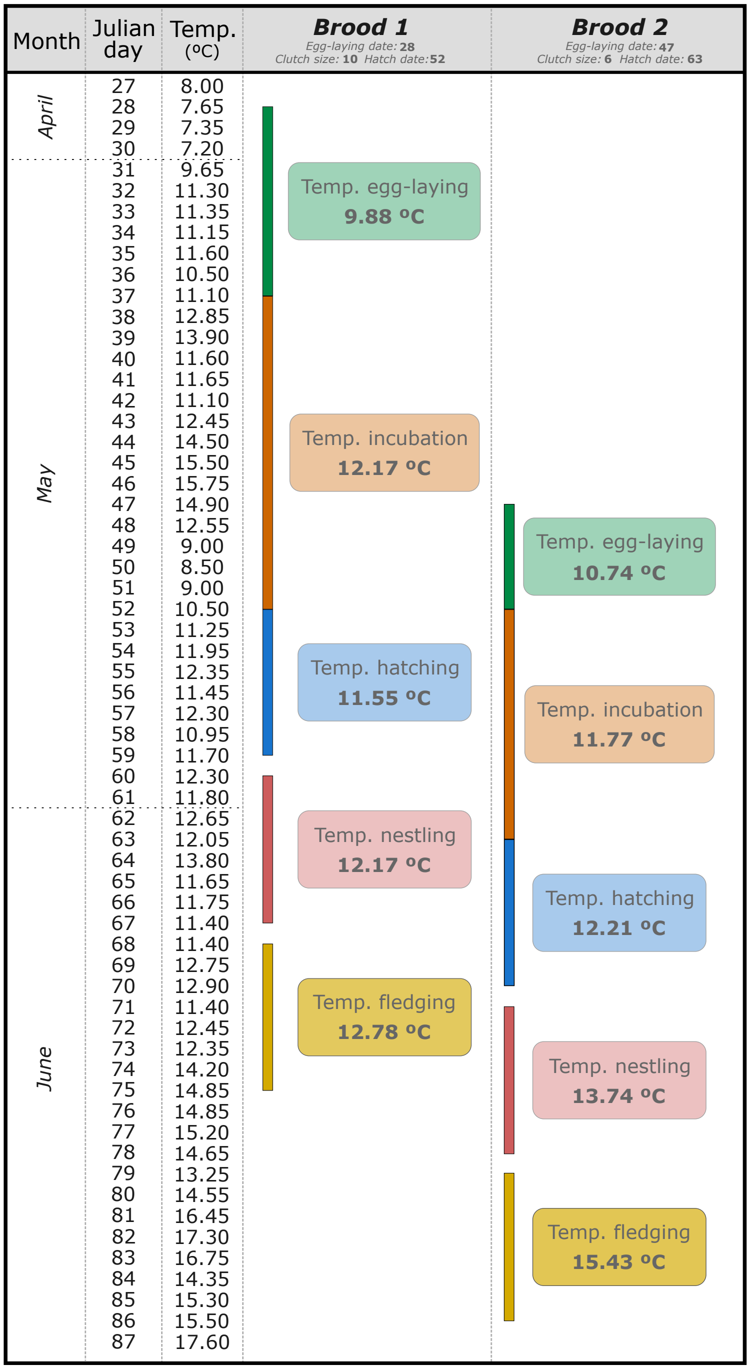

Figure S7. Temporal trends of the hypothetical temperatures experienced by Wytham woods' great tits in the five considered breeding periods. The coloured dots and whiskers represent the yearly mean ± se. The coloured lines and the grey ribbons the temporal trends in temperature in each period. Black lines represent the actual temporal trends in temperature from the models shown in the main text, which were not significant (see the main text for further information on this)

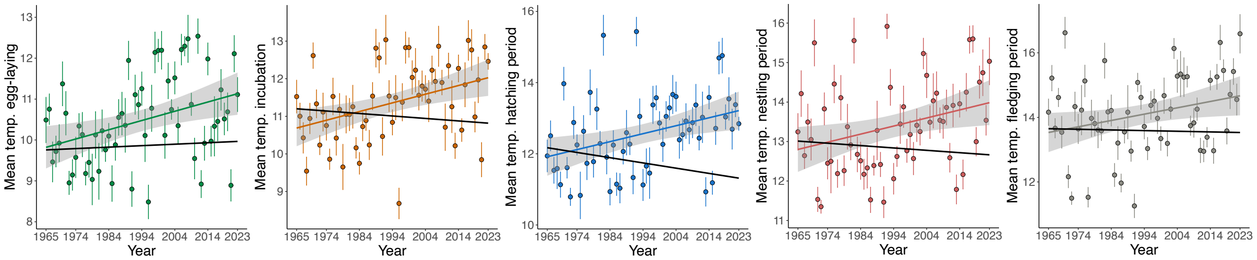

Figure S8: Associations between the within-individual component of temperature and breeding year (i.e. breeding attempt). Black lines and grey ribbons represent the predicted trends of the model and the 95% confidence intervals. Dots represent the raw data. Green, orange, blue, red and yellow represent the egg-laying (**A**), incubation (**B**), hatching (**C**), nestling (**D**) and fledging (**E**) periods respectively.

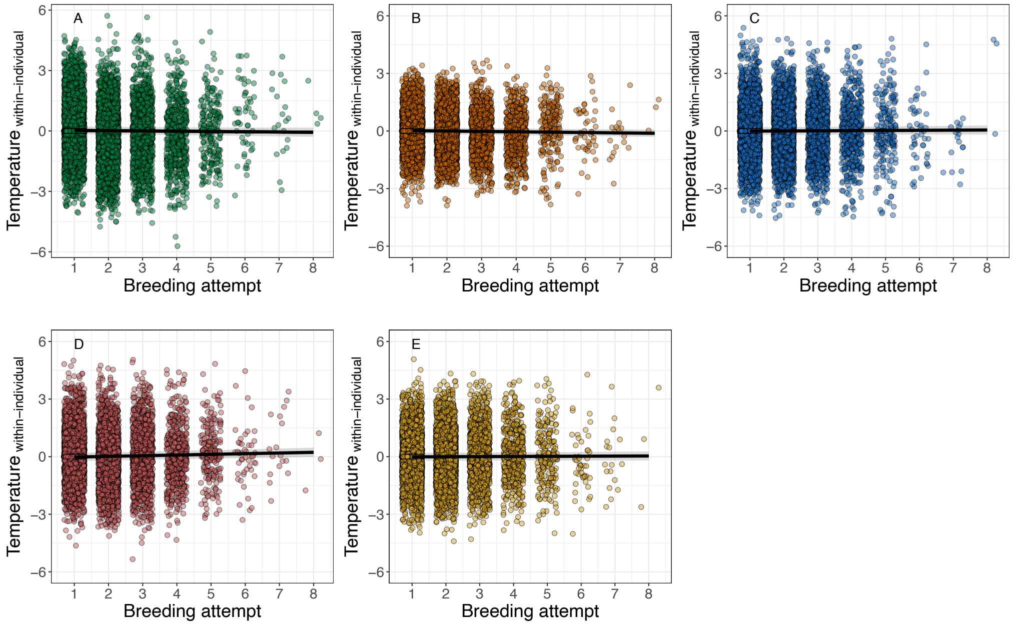

Figure S9: Associations between reproductive success (A: clutch size, B: number of fledglings, C: fledging success and D: number of recruits) and temperature during egg-laying using three analytical approaches: Bayesian model with splines and year as random intercept (1), frequentist model with a random slope and intercept between temperature and year (2) and Bayesian model with splines and year centred reproductive success metrics. Black lines and ribbons represent the trends predicted by the models. Density plots represent the distribution of temperatures at egg-laying. The black dot and horizontal wishers represent the temperature at which reproductive success is maximized and its 95% CIs. The green dot and horizontal wishers the mean temperature of the period ± 1 standard deviation.

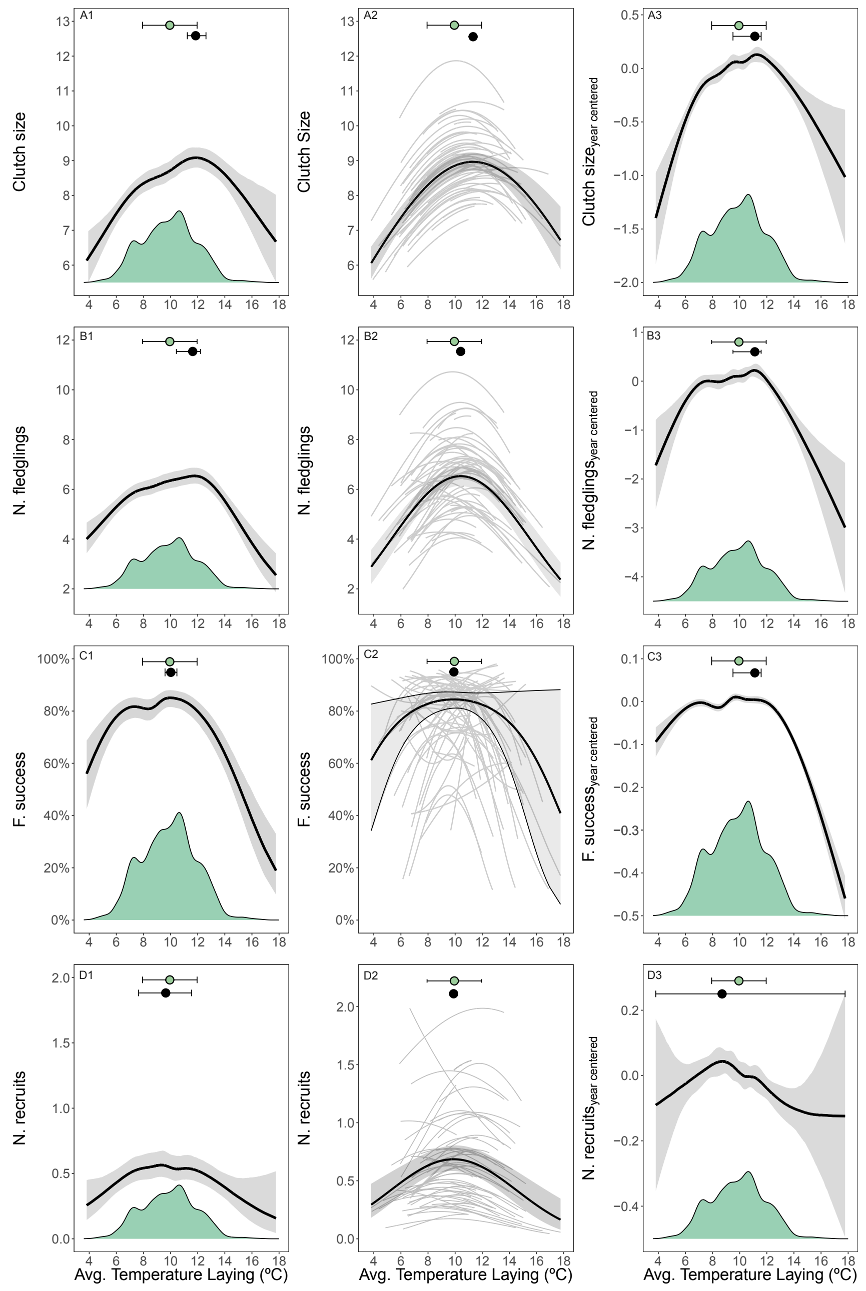

Figure S10: Associations between mismatch with the peak of food availability (half-fall date) and mean temperature during egg-laying using three different analytical methods: **A**) Bayesian model with splines and year as random intercept, **B**) frequentist model including a random slope and intercept between the quadratic component of temperature and year and **C**) Bayesian models with splines and year centered absolute mismatch. The solid line and grey ribbon represent the predicted association and 95% confidence interval from the model. Grey lines in B represent the year-specific trends. Density plots represent the distribution of temperature at egg-laying. The black dot and horizontal wishers represent the temperature at which mismatch is minimised and its 95% CIs. The green dot and horizontal wishers the mean temperature of the period ± 1 standard deviation.

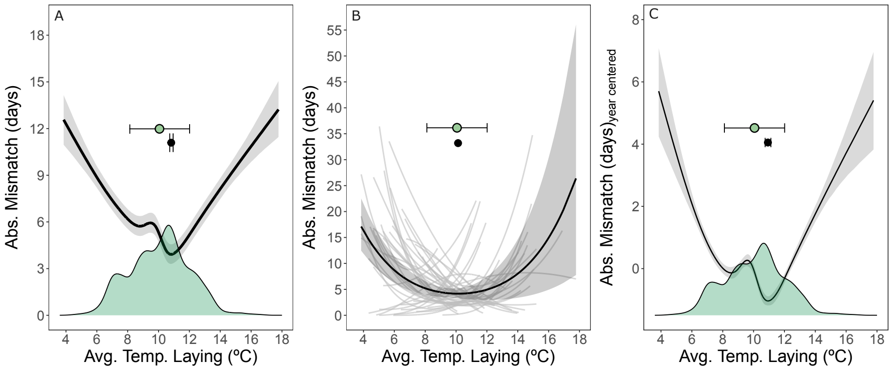

Figure S11: Temporal trends in mismatch between the peak of food availability and peak of food demands between 1965 and 2023. Dots represent the mean ± 1 standard deviation mismatch and the dotted line the association between mismatch and year.

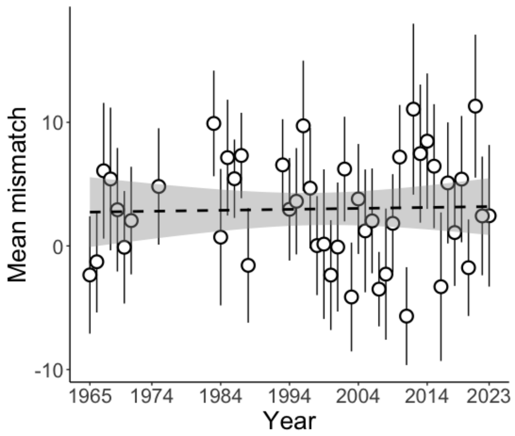

Table S1. Results from the linear mixed models analysing the temporal (i.e. annual) trends in temperature in the five intervals defined relative to individual reproductive timing. Egg-laying represents the period between laying the first and last eggs and incubation the period from the end of egg-laying to hatching. The periods hatching, nestling and fledging capture the time between hatching and 24 days post-hatch in 8-day intervals. The models included female individual identity and year (as a categorical variable) as random effects.

Reproductive Period	Estimate	SE	*F*	*P*
Egg-laying (n=12315)				
Year	0.001	0.009	$F_{1,57.1}$=0.021	0.885
Incubation (n=11643)				
Year	-0.007	0.009	$F_{1,57.1}$=0.646	0.424
Hatching period (n=11643)				
Year	-0.012	0.010	$F_{1,57.1}$=1.313	0.256
Nestling period (n=11643)				
Year	-0.006	0.010	$F_{1,57.1}$=0.456	0.502
Fledging period (n=11643)				
Year	-0.005	0.011	$F_{1,57.0}$=0.281	0.597

Table S2: Results from the model analysing the association between clutch size and temperature at egg-laying. The model included a spline with seven degrees of freedom. Bold denotes statistical support (95% credible interval (CI) not including zero). Fixed effects were scaled to a mean of zero and standard deviation of one. The model included mother identity and year as random factors.

Variable	Estimate	95% CI
Temperatures at egg-laying (n=11033)		
**Mean Temp. 1**	**0.321**	**[0.207, 0.435]**
**Mean Temp. 2**	**0.334**	**[0.206, 0.470]**
**Mean Temp. 3**	**0.358**	**[0.240, 0.484]**
**Mean Temp. 4**	**0.387**	**[0.264, 0.515]**
**Mean Temp. 5**	**0.295**	**[0.206, 0.383]**
**Mean Temp. 6**	**0.472**	**[0.202, 0.749]**
Mean Temp. 7	-0.049	[-0.246, 0.150]
**Laying date**	**-0.097**	**[-0.110, -0.084]**
Num. Neighbours	0.005	[-0.001, 0.012]

Table S3: Results from the model analysing the association between number of fledglings and temperature at egg-laying. The model included a spline with seven degrees of freedom. Bold denotes statistical support (95% credible interval (CI) not including zero). Fixed effects were scaled to a mean of zero and standard deviation of one. The model included mother identity and year as random factors.

Variable	Estimate	95% CI
Temperatures at egg-laying (n=11033)		
**Mean Temp. 1**	**0.413**	**[0.276, 0.551]**
**Mean Temp. 2**	**0.452**	**[0.300, 0.612]**
**Mean Temp. 3**	**0.465**	**[0.320, 0.616]**
**Mean Temp. 4**	**0.482**	**[0.331, 0.639]**
**Mean Temp. 5**	**0.371**	**[0.257, 0.485]**
**Mean Temp. 6**	**0.346**	**[0.007, 0.698]**
**Mean Temp. 7**	**-0.644**	**[-0.956, -0.346]**
**Laying date**	**-0.100**	**[-0.115, -0.086]**
**Num. Neighbours**	**0.013**	**[0.005, 0.021]**

Table S4: Results from the model analysing the association between number of fledglings and temperature at incubation. The model included a spline with seven degrees of freedom. Bold denotes statistical support (95% credible interval (CI) not including zero). Fixed effects were scaled to a mean of zero and standard deviation of one. The model included mother identity and year as random factors.

Variable	Estimate	95% CI
Temperatures incubation (n=10421)		
**Mean Temp. 1**	**0.192**	**[0.091, 0.295]**
**Mean Temp. 2**	**0.250**	**[0.130, 0.370]**
**Mean Temp. 3**	**0.267**	**[0.149, 0.382]**
**Mean Temp. 4**	**0.310**	**[0.193, 0.430]**
**Mean Temp. 5**	**0.188**	**[0.087, 0.288]**
**Mean Temp. 6**	**0.763**	**[0.509, 1.027]**
**Mean Temp. 7**	**0.368**	**[0.180, 0.552]**
**Laying date**	**-0.099**	**[-0.113, -0.086]**
**Num. Neighbours**	**0.014**	**[0.007, 0.022]**

Table S5: Results from the model analysing the association between number of fledglings and temperature during the hatching period. The model included a spline with seven degrees of freedom. Bold denotes statistical support (95% credible interval (CI) not including zero). Fixed effects were scaled to a mean of zero and standard deviation of one. The model included mother identity and year as random factors.

Variable	Estimate	95% CI
Temperatures at hatching (n=10421)		
Mean Temp. 1	0.069	[-0.049, 0.184]
Mean Temp. 2	0.069	[-0.073, 0.207]
Mean Temp. 3	0.038	[-0.094, 0.167]
Mean Temp. 4	0.075	[-0.060, 0.208]
Mean Temp. 5	0.073	[-0.020, 0.167]
Mean Temp. 6	-0.014	[-0.319, 0.282]
Mean Temp. 7	0.040	[-0.126, 0.201]
**Laying date**	**-0.084**	**[-0.096, -0.072]**
**Num. Neighbours**	**0.015**	**[0.007, 0.022]**

Table S6 Results from the model analysing the association between number of fledglings and temperature during the nestling period. The model included a spline with seven degrees of freedom. Bold denotes statistical support (95% credible interval (CI) not including zero). Fixed effects were scaled to a mean of zero and standard deviation of one. The model included mother identity and year as random factors.

Variable	Estimate	95% CI
Temperatures at nestling (n=10421)		
Mean Temp. 1	0.041	[-0.074, 0.155]
Mean Temp. 2	0.037	[-0.098, 0.174]
Mean Temp. 3	0.054	[-0.073, 0.182]
Mean Temp. 4	0.052	[-0.078, 0.180]
Mean Temp. 5	0.056	[-0.035, 0.145]
Mean Temp. 6	0.059	[-0.214, 0.333]
**Mean Temp. 7**	**0.175**	**[0.064, 0.283]**
**Laying date**	**-0.089**	**[-0.102, -0.075]**
**Num. Neighbours**	**0.014**	**[0.006, 0.022]**

Table S7: Results from the model analysing the association between number of fledglings and temperature during the fledging period. The model included a spline with seven degrees of freedom. Bold denotes statistical support (95% credible interval (CI) not including zero). Fixed effects were scaled to a mean of zero and standard deviation of one. The model included mother identity and year as random factors.

Variable	Estimate	95% CI
Temperatures at nestling (n=10421)		
**Mean Temp. 1**	**0.275**	**[0.143, 0.414]**
**Mean Temp. 2**	**0.266**	**[0.112, 0.421]**
**Mean Temp. 3**	**0.306**	**[0.160, 0.462]**
**Mean Temp. 4**	**0.288**	**[0.143, 0.440]**
**Mean Temp. 5**	**0.224**	**[0.127, 0.324]**
**Mean Temp. 6**	**0.537**	**[0.231, 0.865]**
Mean Temp. 7	0.069	[-0.056, 0.194]
**Laying date**	**-0.084**	**[-0.096, -0.071]**
**Num. Neighbours**	**0.014**	**[0.006, 0.022]**

Table S8: Results from the model analysing the association between fledging success and temperature at egg-laying. The model included a spline with seven degrees of freedom. Bold denotes statistical support (95% credible interval (CI) not including zero). Fixed effects were scaled to a mean of zero and standard deviation of one. The model included mother identity and year as random factors.

Variable	Estimate	95% CI
Temperatures at egg-laying (n=11033)		
**Mean Temp. 1**	**1.099**	**[0.606, 1.593]**
**Mean Temp. 2**	**1.527**	**[0.956, 2.083]**
**Mean Temp. 3**	**1.504**	**[0.973, 2.037]**
**Mean Temp. 4**	**1.405**	**[0.858, 1.934]**
Mean Temp. 5	0.040	[-0.347, 0.430]
Mean Temp. 6	0.724	[-0.453, 1.857]
**Mean Temp. 7**	**-2.420**	**[-3.134, -1.689]**
Laying date	-0.050	[-0.112, 0.011]
**Clutch size**	**-0.064**	**[-0.103, -0.024]**
Num. Neighbours	0.010	[-0.023, 0.042]

Table S9: Results from the model analysing the association between fledging success and temperature during incubation. The model included a spline with seven degrees of freedom. Bold denotes statistical support (95% credible interval (CI) not including zero). Fixed effects were scaled to a mean of zero and standard deviation of one. The model included mother identity and year as random factors.

Variable	Estimate	95% CI
Temperatures at incubation (n=10421)		
**Mean Temp. 1**	**0.467**	**[0.129, 0.819]**
**Mean Temp. 2**	**0.854**	**[0.452, 1.251]**
**Mean Temp. 3**	**0.896**	**[0.502, 1.285]**
**Mean Temp. 4**	**0.909**	**[0.500, 1.328]**
Mean Temp. 5	0.140	[-0.245, 0.530]
**Mean Temp. 6**	**1.904**	**[1.048, 2.760]**
Mean Temp. 7	0.101	[-0.596, 0.816]
**Laying date**	**-0.193**	**[-0.250, -0.134]**
**Clutch size**	**-0.090**	**[-0.128, -0.050]**
Num. Neighbours	0.003	[-0.030, 0.038]

Table S10: Results from the model analysing the association between fledging success and temperature during the hatching periods. The model included a spline with seven degrees of freedom. Bold denotes statistical support (95% credible interval (CI) not including zero). Fixed effects were scaled to a mean of zero and standard deviation of one. The model included mother identity and year as random factors.

Variable	Estimate	95% CI
Temperatures at hatching (n=10421)		
**Mean Temp. 1**	**1.006**	**[0.569, 1.428]**
**Mean Temp. 2**	**0.794**	**[0.276, 1.305]**
**Mean Temp. 3**	**0.779**	**[0.301, 1.254]**
**Mean Temp. 4**	**0.785**	**[0.286, 1.284]**
**Mean Temp. 5**	**0.603**	**[0.243, 0.966]**
Mean Temp. 6	0.693	[-0.390, 1.792]
Mean Temp. 7	-0.276	[-0.916, 0.369]
**Laying date**	**-0.185**	**[-0.240, -0.129]**
**Clutch size**	**-0.090**	**[-0.129, -0.052]**
Num. Neighbours	0.004	[-0.029, 0.038]

Table S11: Results from the model analysing the association between fledging success and temperature during the nestling period. The model included a spline with seven degrees of freedom. Bold denotes statistical support (95% credible interval (CI) not including zero). Fixed effects were scaled to a mean of zero and standard deviation of one. The model included mother identity and year as random factors.

Variable	Estimate	95% CI
Temperatures at nestling (n=10421)		
Mean Temp. 1	0.291	[-0.149, 0.729]
Mean Temp. 2	0.301	[-0.235, 0.807]
Mean Temp. 3	0.008	[-0.485, 0.502]
Mean Temp. 4	0.296	[-0.213, 0.788]
Mean Temp. 5	-0.062	[-0.386, 0.285]
Mean Temp. 6	0.359	[-0.712, 1.416]
**Mean Temp. 7**	**0.863**	**[0.402, 1.338]**
**Laying date**	**-0.179**	**[-0.234, -0.123]**
**Clutch size**	**-0.089**	**[-0.127, -0.050]**
Num. Neighbours	0.007	[-0.026, 0.039]

Table S12 Results from the model analysing the association between fledging success and temperature during the fledgling period. The model included a spline with seven degrees of freedom. Bold denotes statistical support (95% credible interval (CI) not including zero). Fixed effects were scaled to a mean of zero and standard deviation of one. The model included mother identity and year as random factors.

Variable	Estimate	95% CI
Temperatures at nestling (n=10421)		
**Mean Temp. 1**	**0.677**	**[0.238, 1.094]**
**Mean Temp. 2**	**1.062**	**[0.556, 1.554]**
**Mean Temp. 3**	**0.743**	**[0.245, 1.202]**
**Mean Temp. 4**	**0.622**	**[0.130, 1.095]**
Mean Temp. 5	0.230	[-0.105, 0.557]
Mean Temp. 6	1.261	[0.270, 2.229]
Mean Temp. 7	-0.171	[-0.652, 0.336]
Laying date	-0.150	[-0.203, -0.097]
Clutch size	-0.085	[-0.123, -0.045]
Num. Neighbours	0.007	[-0.027, 0.040]

Table S13: Results from the model analysing the association between number of recruits and temperature at egg-laying. The model included a spline with seven degrees of freedom. Bold denotes statistical support (95% credible interval (CI) not including zero). Fixed effects were scaled to a mean of zero and standard deviation of one. The model included mother identity and year as random factors.

Variable	Estimate	95% CI
Temperatures at fledgling (n=11033)		
**Mean Temp. 1**	**0.757**	**[0.258, 1.274]**
**Mean Temp. 2**	**0.819**	**[0.233, 1.416]**
**Mean Temp. 3**	**0.717**	**[0.174, 1.265]**
**Mean Temp. 4**	**0.768**	**[0.199, 1.340]**
Mean Temp. 5	0.331	[-0.090, 0.771]
Mean Temp. 6	0.691	[-0.269, 2.022]
Mean Temp. 7	-0.831	[-2.118, 0.427]
**Laying date**	**-0.038**	**[-0.045, -0.032]**
**Num. Neighbours**	**0.054**	**[0.037, 0.072]**

Table S14 Results from the model analysing the association between number of recruits and temperature during incubation. The model included a spline with seven degrees of freedom. Bold denotes statistical support (95% credible interval (CI) not including zero). Fixed effects were scaled to a mean of zero and standard deviation of one. The model included mother identity and year as random factors.

Variable	Estimate	95% CI
Temperatures at incubation (n=10421)		
Mean Temp. 1	0.099	[-0.272, 0.480]
Mean Temp. 2	0.205	[-0.212, 0.640]
Mean Temp. 3	0.384	[-0.018, 0.809]
Mean Temp. 4	0.171	[-0.250, 0.595]
**Mean Temp. 5**	**0.443**	**[0.080, 0.830]**
Mean Temp. 6	0.289	[-0.674, 1.226]
**Mean Temp. 7**	**-1.030**	**[-1.863, -0.224]**
**Laying date**	**-0.039**	**[-0.045, -0.034]**
**Num. Neighbours**	**0.056**	**[0.038, 0.073]**

Table S15: Results from the model analysing the association between number of recruits and temperature during hatching. The model included a spline with seven degrees of freedom. Bold denotes statistical support (95% credible interval (CI) not including zero). Fixed effects were scaled to a mean of zero and standard deviation of one. The model included mother identity and year as random factors.

Variable	Estimate	95% CI
Temperatures at hatching (n=10421)		
Mean Temp. 1	0.125	[-0.251, 0.499]
Mean Temp. 2	0.355	[-0.087, 0.797]
Mean Temp. 3	0.105	[-0.315, 0.516]
Mean Temp. 4	0.237	[-0.182, 0.659]
Mean Temp. 5	0.129	[-0.173, 0.445]
Mean Temp. 6	0.228	[-0.739, 1.182]
Mean Temp. 7	0.049	[-0.545, 0.660]
**Laying date**	**-0.331**	**[-0.377, -0.285]**
**Num. Neighbours**	**0.086**	**[0.059, 0.112]**

Table S16: Results from the model analysing the association between number of recruits and temperature during the nestling period. The model included a spline with seven degrees of freedom. Bold denotes statistical support (95% credible interval (CI) not including zero). Fixed effects were scaled to a mean of zero and standard deviation of one. The model included mother identity and year as random factors.

Variable	Estimate	95% CI
Temperatures at nestling (n=10421)		
Mean Temp. 1	0.112	[-0.233, 0.455]
Mean Temp. 2	0.368	[-0.028, 0.782]
Mean Temp. 3	0.080	[-0.292, 0.453]
Mean Temp. 4	0.206	[-0.172, 0.580]
Mean Temp. 5	-0.103	[-0.415, 0.212]
Mean Temp. 6	0.480	[-0.304, 1.274]
Mean Temp. 7	0.162	[-0.170, 0.504]
**Laying date**	**-0.314**	**[-0.361, -0.266]**
Num. Neighbours	0.086	[-0.061, 0.113]

Table S17: Results from the model analysing the association between number of recruits and temperature during the fledging period. The model included a spline with seven degrees of freedom. Bold denotes statistical support (95% credible interval (CI) not including zero). Fixed effects were scaled to a mean of zero and standard deviation of one. The model included mother identity and year as random factors.

Variable	Estimate	95% CI
Temperatures at fledging (n=10421)		
Mean Temp. 1	0.423	[-0.008, 0.863]
**Mean Temp. 2**	**0.681**	**[0.189, 1.207]**
Mean Temp. 3	0.401	[-0.056, 0.878]
**Mean Temp. 4**	**0.556**	**[0.071, 1.051]**
Mean Temp. 5	0.228	[-0.082, 0.547]
**Mean Temp. 6**	**1.307**	**[0.320, 2.345]**
Mean Temp. 7	0.061	[-0.315, 0.426]
**Laying date**	**-0.318**	**[-0.365, -0.272]**
**Num. Neighbours**	**0.086**	**[0.060, 0.113]**

Table S18: Results from the linear mixed models with natural cubic splines (df=7) analysing the association between absolute mismatch with winter-moth half-fall dates and temperature at the periods relative to breeding. Bold denotes model support (95% credible intervals (CI) including zero) The model included mother identity and year as random factors. Sample size is smaller in this set of models as half-fall dates were available for 45 of the 59 years.

Variable	Estimate	95% CI
Temperatures at egg-laying (n=9711)		
**Mean Temp. 1**	**-7.192**	**[-8.524, -5.739]**
**Mean Temp. 2**	**-6.117**	**[-7.792, -4.432]**
**Mean Temp. 3**	**-8.820**	**[-10.248, -7.309]**
**Mean Temp. 4**	**-8.488**	**[-10.052, -6.965]**
**Mean Temp. 5**	**-2.559**	**[-3.548, -1.570]**
**Mean Temp. 6**	**-6.464**	**[-9.594, -3.177]**
**Mean Temp. 7**	**3.209**	**[1.484, 4.917]**
Temperatures at incubation (n=9711)		
**Mean Temp. 1**	**-13.433**	**[-14.262, -12.598]**
**Mean Temp. 2**	**-15.864**	**[-16.810, -14.899]**
**Mean Temp. 3**	**-17.194**	**[-18.112, -16.252]**
**Mean Temp. 4**	**-15.993**	**[-16.913, -15.023]**
**Mean Temp. 5**	**-12.374**	**[-13.173, -11.596]**
**Mean Temp. 6**	**-34.349**	**[-36.287, -32, 353]**
**Mean Temp. 7**	**-7.623**	**[-8.882, -6.427]**
Temperatures at hatching (n=9711)		
**Mean Temp. 1**	**-4.611**	**[-5.731, -3.552]**
**Mean Temp. 2**	**-4.937**	**[-6.260, -3.647]**
**Mean Temp. 3**	**-5.223**	**[-6.455, -4.095]**
**Mean Temp. 4**	**-5.106**	**[-6.372, -3.900]**
**Mean Temp. 5**	**-8.308**	**[-9.185, -7.360]**
**Mean Temp. 6**	**-3.932**	**[-6.768, -1.288]**
**Mean Temp. 7**	**2.022**	**[0.312, 3.641]**
Temperatures at nestling (n=9711)		
**Mean Temp. 1**	**-9.061**	**[-10.096, -8.095]**
**Mean Temp. 2**	**-10.853**	**[-12.192, -9.686]**
**Mean Temp. 3**	**-9.357**	**[-10.513, -8.281]**
**Mean Temp. 4**	**-10.868**	**[-12.043, -9.735]**
**Mean Temp. 5**	**-7.793**	**[-8.614, -7.022]**
**Mean Temp. 6**	**-12.430**	**[-15.008, -9.860]**
**Mean Temp. 7**	**-7.870**	**[-9.100, -6.619]**

Table S19 Estimates and 95% credible intervals (CI) from the pre- and post-peak models analysing the association between absolute mismatch and temperature during the four relative periods. Bold denotes model support (95% credible intervals (CI) including zero) The model included mother identity and year as random factors. Sample size is smaller in this set of models as half-fall dates were available for 45 of the 59 years.

Period	Estimates [95% CIs]	
	Pre-peak	Post-peak
Egg-laying	**-0.995 [-1.113, -0.873]**	**1.259 [1.139, 1.375]**
Incubation	**-1.944 [-2.085, -1.808]**	0.166 [-0.060, 0.389]
Hatching	**-1.342 [-1.443, -1.241]**	**1.024 [0.842, 1.211]**
Nestling	**-1.834 [-1.920, -1.747]**	**0.418 [0.273, 0.562]**

Table S20: Results from the model analysing the association between reproductive success and pre and post-peak temperatures at egg-laying including and not including. These models had the same structure as those presented in the main text, but note that the estimates in these differ from those in the main text due to the sample size reduction required to include mismatch (see methods).

	Clutch size		N. Fledglings	
	No mismatch	With mismatch	No mismatch	With mismatch
Pre-peak	0.052 [0.040, 0.063]	0.053 [0.041, 0.065]	0.049 [0.033, 0.066]	0.048 [0.032, 0.065]
Post-peak	-0.077 [-0.106, -0.048]	-0.081 [-0.110, -0.053]	-0.048 [-0.067, -0.030]	-0.027 [-0.046, -0.008]
	**Fledging Success**		**N. Recruits**	
	No mismatch	With mismatch	No mismatch	With mismatch
Pre-peak	0.063 [-0.002, 0.128]	0.071 [0.004, 0.139]	0.101 [0.035, 0.164]	0.101 [0.034, 0.166]
Post-peak	-0.297 [-0.387, -0.204]	-0.142 [-0.237, -0.048]	-0.037 [-0.097, 0.024]	-0.009 [-0.068, 0.053]

Table S21: The change in reproductive success with post-peak temperatures is notably reduced when including mismatch. Values represent the mean and 95% credible intervals change with temperatures higher than the peak including and not mismatch. Values are in the units of the reproductive trait.

	N. Fledglings		Fledging Success		N. Recruits	
	Post-peak no mismatch	Post-peak w. mismatch	Post-peak no mismatch	Post-peak w. mismatch	Post-peak no mismatch	Post-peak w. mismatch
Laying	-2.06 [-2.76, -1.32]	-1.22 [-2.01, -0.39]	-34.30 [-49.12, -20.18]	-13.40 [-26.22, -3.60]	-0.13 [-0.34, 0.10]	-0.03 [-0.24, 0.24]

Table S22: Estimates ± 95 credible intervals (CI) of the pre and post peak trends obtained from the three modelling approaches. Empty cells correspond to models in which the peak of temperature was the maximum, no post-peak trends were fitted. Bold denotes model support (95% CI not including zero).

Period	Splines (df=7)		Splines (df=5)		Splines (df=9)	
	Clutch size					
	Pre-peak	Post-peak	Pre-peak	Post-peak	Pre-peak	Post-peak
Egg-laying	**0.053 [0.042, 0.064]**	**-0.059 [-0.081, -0.038]**	**0.053 [0.042, 0.064]**	**-0.059 [-0.081, -0.038]**	**0.053 [0.042, 0.064]**	**-0.059 [-0.080, -0.039]**
	Number of fledglings					
	Pre-peak	Post-peak	Pre-peak	Post-peak	Pre-peak	Post-peak
Egg-laying	**0.050 [0.037, 0.063]**	**-0.087 [-0.113, -0.061]**	**0.049 [0.036, 0.062]**	**-0.087 [-0.111, -0.062]**	**0.051 [0.039, 0.064]**	**-0.066 [-0.087, -0.044]**
Incubation	**0.042 [0.027, 0.057]**	-	**0.042 [0.027, 0.057]**	-	**0.043 [0.028, 0.058]**	*Only 5 obs.*
Hatching	**0.021 [0.007, 0.034]**	-0.003 [-0.025, 0.019]	0.021 [0.009, 0.032]	-0.007 [-0.042, 0.029]	**0.020 [0.008, 0.033]**	-0.013 [-0.044, 0.019]
Nestling	**0.024 [0.012, 0.035]**	-	**0.024 [0.012, 0.035]**	-	**0.024 [0.012, 0.035]**	-
Fledgling	**0.021 [0.009, 0.034]**	-0.002 [-0.074, 0.079]	0.022 [0.010, 0.034]	-0.024 [-0.081, 0.032]	**0.022 [0.010, 0.035]**	0.007 [-0.070, 0.088]
	Fledgling success					
	Pre-peak	Post-peak	Pre-peak	Post-peak	Pre-peak	Post-peak
Egg-laying	-0.006 [-0.074, 0.061]	**-0.310 [-0.385, -0.234]**	**-0.006 [-0.063, 0.054]**	**-0.313 [-0.403, -0.221]**	**0.011 [-0.049, 0.068]**	**-0.321 [-0.415, 0.233]**
Incubation	-0.032 [-0.098, 0.036]	**0.112 [0.023, 0.205]**	0.045 [-0.017, 0.105]	0.188 [-0.022, 0.413]	0.020 [-0.038, 0.076]	*Only 11 obs.*
Hatching	0.009 [-0.101, 0.119]	**-0.094 [-0.148, -0.041]**	0.055 [-0.050, 0.158]	**-0.103 [-0.163, -0.041]**	0.010 [-0.094, 0.124]	**-0.110 [-0.164, -0.058]**
Nestling	0.038 [-0.004, 0.081]	-	0.038 [-0.004, 0.081]	-	0.038 [-0.004, 0.081]	-
Fledgling	**0.094 [0.021, 0.167]**	**-0.074 [-0.136, -0.009]**	0.063 [-0.018, 0.145]	**-0.079 [-0.138, -0.017]**	**0.076 [0.006, 0.146]**	**-0.063 [-0.126, -0.002]**
	Number of recruits					
	Pre-peak	Post-peak	Pre-peak	Post-peak	Pre-peak	Post-peak
Egg-laying	**0.093 [0.037, 0.148]**	-0.026 [-0.082, 0.030]	**0.099 [0.043, 0.153]**	-0.033 [-0.091, 0.021]	**0.098 [0.044, 0.154]**	-0.021 [-0.082, 0.038]
Incubation	-0.004 [-0.059, 0.050]	0.064 [-0.004, 0.131]	-0.015 [-0.074, 0.043]	0.045 [-0.020, 0.106]	-0.008 [-0.061, 0.046]	0.060 [-0.010, 0.136]
Hatching	0.039 [-0.007, 0.084]	-0.070 [-0.148, 0.008]	0.049 [-0.005, 0.101]	-0.023 [-0.083, 0.036]	0.041 [-0.012, 0.094]	-0.025 [-0.083, 0.034]
Nestling	**-0.011 [-0.053, 0.030]**	-	**-0.011 [-0.053, 0.030]**	-	**-0.011 [-0.053, 0.030]**	-
Fledgling	0.104 [-0.011, 0.225]	-0.009 [-0.052, 0.033]	-0.015 [-0.066, 0.036]	-0.012 [-0.060, 0.037]	0.001 [-0.066, 0.067]	-0.014 [-0.061, 0.031]

Table S23: Result from the linear model analysing the temporal trends in mean temperature during the fixed interval with the reduced dataset.

Variable	Estimate	Std. Error	z	P
		Fixed window		
**Mean Temp.**	**0.035**	**0.006**	**$F_{1,43}$=25.21**	**<0.001**

Table S24 Result from the linear mixed models analysing the temporal trends in temperature in the five periods defined relative to individual reproductive timing using the reduced dataset. Egg-laying represents the period between laying the first and last eggs and incubation the period from the end of egg-laying to hatching. The periods hatching, nestling and fledging capture the time between hatching and 24 days post-hatch in 8-day intervals. The models included female individual identity and year (as a categorical variable) as random effects.

Reproductive Period	Estimate	SE	*F*	*P*
		Egg-laying		
Year	-0.012	0.010	$F_{1,43.2}$=1.308	0.258
		Incubation		
Year	-0.007	0.011	$F_{1,43.0}$=0.400	0.529
		Hatching period		
Year	0.0004	0.010	$F_{1,43}$=0.002	0.964
		Nestling period		
Year	-0.006	0.010	$F_{1,43.1}$=0.380	0.540
		Fledging period		
Year	-0.012	0.011	$F_{1,43.0}$=1.669	0.286

Table S25: Results from the model analysing the association between clutch size and temperature at egg-laying using the reduced dataset. The model included a spline with seven degrees of freedom. Bold denotes statistical support (95% credible interval (CI) not including zero). Fixed effects were scaled to a mean of zero and standard deviation of one. The model included mother identity and year as random factors.

Variable	Estimate	95% CI
	Temperatures at egg-laying	
**Mean Temp. 1**	**0.377**	**[0.229, 0.537]**
**Mean Temp. 2**	**0.388**	**[0.220, 0.561]**
**Mean Temp. 3**	**0.432**	**[0.273, 0.592]**
**Mean Temp. 4**	**0.440**	**[0.277, 0.606]**
**Mean Temp. 5**	**0.330**	**[0.220, 0.438]**
**Mean Temp. 6**	**0.586**	**[0.227, 0.939]**
Mean Temp. 7	-0.037	[-0.242, 0.176]
**Laying date**	**-0.094**	**[-0.108, -0.081]**
Num. Neighbours	0.006	[-0.001, 0.014]

Table S26: Results from the model analysing the association between number of fledglings and temperature at egg-laying using the reduced dataset. The model included a spline with seven degrees of freedom. Bold denotes statistical support (95% credible interval (CI) not including zero). Fixed effects were scaled to a mean of zero and standard deviation of one. The model included mother identity and year as random factors.

Variable	Estimate	95% CI
Temperatures at egg-laying		
**Mean Temp. 1**	**0.454**	**[0.269, 0.633]**
**Mean Temp. 2**	**0.470**	**[0.250, 0.675]**
**Mean Temp. 3**	**0.515**	**[0.311, 0.709]**
**Mean Temp. 4**	**0.507**	**[0.309, 0.718]**
**Mean Temp. 5**	**0.394**	**[0.236, 0.540]**
Mean Temp. 6	0.384	[-0.078, 0.850]
**Mean Temp. 7**	**-0.662**	**[-1.030, -0.308]**
**Laying date**	**-0.102**	**[-0.118, -0.086]**
**Num. Neighbours**	**0.018**	**[0.010, 0.026]**

Table S27: Results from the model analysing the association between fledging success and temperature at egg-laying using the reduced dataset. The model included a spline with seven degrees of freedom. Bold denotes statistical support (95% credible interval (CI) not including zero). Fixed effects were scaled to a mean of zero and standard deviation of one. The model included mother identity and year as random factors.

Variable	Estimate	95% CI
Temperatures at egg-laying		
**Mean Temp. 1**	**1.619**	**[1.042, 2.207]**
**Mean Temp. 2**	**1.864**	**[1.223, 2.534]**
**Mean Temp. 3**	**2.013**	**[1.414, 2.642]**
**Mean Temp. 4**	**1.847**	**[1.226, 2.495]**
Mean Temp. 5	0.246	[-0.203, 0.685]
**Mean Temp. 6**	**1.453**	**[0.117, 2.837]**
**Mean Temp. 7**	**-2.084**	**[-2.871, -1.313]**
**Laying date**	**-0.107**	**[-0.175, -0.037]**
**Clutch size**	**-0.131**	**[-0.174, -0.088]**
Num. Neighbours	0.018	[-0.017, 0.055]

Table S28: Results from the model analysing the association between number of recruits and temperature at egg-laying using the reduced dataset. The model included a spline with seven degrees of freedom. Bold denotes statistical support (95% credible interval (CI) not including zero). Fixed effects were scaled to a mean of zero and standard deviation of one. The model included mother identity and year as random factors.

Variable	Estimate	95% CI
Temperatures at agg-laying		
Mean Temp. 1	0.530	[-0.078, 1.263]
Mean Temp. 2	0.561	[-0.160, 1.338]
Mean Temp. 3	0.441	[-0.228, 1.219]
Mean Temp. 4	0.557	[-0.133, 1.361]
Mean Temp. 5	0.156	[-0.361, 0.702]
Mean Temp. 6	0.057	[-1.573, 1.791]
Mean Temp. 7	-0.775	[-2.152, 0.549]
**Laying date**	**-0.344**	**[-0.403, -0.286]**
**Num. Neighbours**	**0.090**	**[0.058, 0.119]**

Table S29: Results from the model analysing the association between number of fledglings and temperature at incubation using the reduced dataset. The model included a spline with seven degrees of freedom. Bold denotes statistical support (95% credible interval (CI) not including zero). Fixed effects were scaled to a mean of zero and standard deviation of one. The model included mother identity and year as random factors.

Variable	Estimate	95% CI
Temperatures incubation		
**Mean Temp. 1**	**0.224**	**[0.111, 0.334]**
**Mean Temp. 2**	**0.267**	**[0.129, 0.339]**
**Mean Temp. 3**	**0.283**	**[0.155, 0.407]**
**Mean Temp. 4**	**0.318**	**[0.187, 0.446]**
**Mean Temp. 5**	**0.173**	**[0.068, 0.271]**
**Mean Temp. 6**	**0.832**	**[0.549, 1.106]**
**Mean Temp. 7**	**0.399**	**[0.236, 0.559]**
**Laying date**	**-0.102**	**[-0.117, -0.087]**
**Num. Neighbours**	**0.019**	**[0.010, 0.028]**

Table S30: Results from the model analysing the association between fledging success and temperature during incubation using the reduced dataset. The model included a spline with seven degrees of freedom. Bold denotes statistical support (95% credible interval (CI) not including zero). Fixed effects were scaled to a mean of zero and standard deviation of one. The model included mother identity and year as random factors.

Variable	Estimate	95% CI
Temperatures at incubation		
**Mean Temp. 1**	**0.489**	**[0.105, 0.863]**
**Mean Temp. 2**	**0.891**	**[0.463, 1.301]**
**Mean Temp. 3**	**0.842**	**[0.410, 1.254]**
**Mean Temp. 4**	**0.946**	**[0.503, 1.355]**
Mean Temp. 5	0.199	[-0.194, 0.590]
**Mean Temp. 6**	**2.333**	**[1.417, 3.186]**
Mean Temp. 7	0.418	[-0.155, 0.944]
**Laying date**	**-0.228**	**[-0.293. -0.162]**
**Clutch size**	**-0.132**	**[-0.173, -0.088]**
Num. Neighbours	0.010	[-0.027, 0.048]

Table S31: Results from the model analysing the association between number of recruits and temperature during incubation using the reduced dataset. The model included a spline with seven degrees of freedom. Bold denotes statistical support (95% credible interval (CI) not including zero). Fixed effects were scaled to a mean of zero and standard deviation of one. The model included mother identity and year as random factors.

Variable	Estimate	95% CI
Temperatures at incubation		
Mean Temp. 1	-0.003	[-0.420, 0.390]
Mean Temp. 2	0.116	[-0.353, 0.575]
Mean Temp. 3	0.332	[-0.132, 0.784]
Mean Temp. 4	0.069	[-0.400, 0.519]
**Mean Temp. 5**	**0.512**	**[0.117, 0.898]**
Mean Temp. 6	0.451	[-0.539, 1.389]
**Mean Temp. 7**	**-0.663**	**[-1.361, -0.006]**
**Laying date**	**-0.351**	**[-0.406, -0.297]**
**Num. Neighbours**	**0.089**	**[0.060, 0.119]**

Table S32: Results from the model analysing the association between number of fledglings and temperature during the hatching period using the reduced dataset. The model included a spline with seven degrees of freedom. Bold denotes statistical support (95% credible interval (CI) not including zero). Fixed effects were scaled to a mean of zero and standard deviation of one. The model included mother identity and year as random factors.

Variable	Estimate	95% CI
Temperatures at hatching		
Mean Temp. 1	0.069	[-0.063, 0.198]
Mean Temp. 2	0.078	[-0.068, 0.221]
Mean Temp. 3	0.047	[-0.092, 0.192]
Mean Temp. 4	0.072	[-0.073, 0.215]
Mean Temp. 5	0.090	[-0.024, 0.208]
Mean Temp. 6	-0.070	[-0.409, 0.248]
Mean Temp. 7	-0.103	[-0.367, 0.136]
**Laying date**	**-0.092**	**[-0.106, -0.077]**
**Num. Neighbours**	**0.019**	**[0.011, 0.028]**

Table S33: Results from the model analysing the association between fledging success and temperature during the hatching periods using the reduced dataset. The model included a spline with seven degrees of freedom. Bold denotes statistical support (95% credible interval (CI) not including zero). Fixed effects were scaled to a mean of zero and standard deviation of one. The model included mother identity and year as random factors.

Variable	Estimate	95% CI
Temperatures at hatching		
**Mean Temp. 1**	**0.989**	**[0.500, 1.446]**
**Mean Temp. 2**	**0.800**	**[0.253, 1.345]**
**Mean Temp. 3**	**0.771**	**[0.257, 1.288]**
**Mean Temp. 4**	**0.855**	**[0.331, 1.363]**
Mean Temp. 5	0.261	[-0.137, 0.645]
Mean Temp. 6	1.068	[-0.100. 2.180]
Mean Temp. 7	0.736	[-0.050, 1.591]
**Laying date**	**-0.214**	**[-0.276, -0.150]**
**Clutch size**	**-0.125**	**[-0.165, -0.083]**
Num. Neighbours	0.008	[-0.028, 0.044]

Table S34: Results from the model analysing the association between number of recruits and temperature during hatching using the reduced dataset. The model included a spline with seven degrees of freedom. Bold denotes statistical support (95% credible interval (CI) not including zero). Fixed effects were scaled to a mean of zero and standard deviation of one. The model included mother identity and year as random factors.

Variable	Estimate	95% CI
Temperatures at hatching		
Mean Temp. 1	0.173	[-0.239, 0.604]
**Mean Temp. 2**	**0.480**	**[0.014, 0.959]**
Mean Temp. 3	0.203	[-0.216, 0.690]
Mean Temp. 4	0.396	[-0.065, 0.882]
Mean Temp. 5	-0.152	[-0.527, 0.236]
Mean Temp. 6	0.849	[-0.180, 1.960]
Mean Temp. 7	0.904	[-0.001, 1.850]
**Laying date**	**-0.337**	**[-0.389, -0.285]**
**Num. Neighbours**	**0.090**	**[0.060, 0.120]**

Table S35: Results from the model analysing the association between number of fledglings and temperature during the nestling period using the reduced dataset. The model included a spline with seven degrees of freedom. Bold denotes statistical support (95% credible interval (CI) not including zero). Fixed effects were scaled to a mean of zero and standard deviation of one. The model included mother identity and year as random factors.

Variable	Estimate	95% CI
Temperatures at nestling		
Mean Temp. 1	0.062	[-0.049, 0.178]
Mean Temp. 2	0.071	[-0.063, 0.212]
Mean Temp. 3	0.073	[-0.047, 0.198]
Mean Temp. 4	0.082	[-0.045, 0.221]
Mean Temp. 5	0.089	[-0.008, 0.193]
Mean Temp. 6	0.069	[-0.204, 0.339]
**Mean Temp. 7**	**0.200**	**[0.028, 0.373]**
**Laying date**	**-0.105**	**[-0.122, -0.088]**
**Num. Neighbours**	**0.019**	**[0.010, 0.028]**

Table S36: Results from the model analysing the association between fledging success and temperature during the nestling period using the reduced dataset. The model included a spline with seven degrees of freedom. Bold denotes statistical support (95% credible interval (CI) not including zero). Fixed effects were scaled to a mean of zero and standard deviation of one. The model included mother identity and year as random factors.

Variable	Estimate	95% CI
Temperatures at nestling		
**Mean Temp. 1**	**0.492**	**[0.010, 0.964]**
Mean Temp. 2	0.558	[-0.009, 1.083]
Mean Temp. 3	0.079	[-0.453, 0.579]
**Mean Temp. 4**	**0.565**	**[0.031, 1.078]**
Mean Temp. 5	-0.118	[-0.524, 0.287]
Mean Temp. 6	0.641	[-0.468, 1.747]
**Mean Temp. 7**	**1.218**	**[0.558, 1.869]**
**Laying date**	**-0.026**	**[-0.034, -0.018]**
**Clutch size**	**-0.076**	**[-0.103, -0.052]**
Num. Neighbours	0.008	[-0.017, 0.033]

Table S37: Results from the model analysing the association between number of recruits and temperature during the nestling period using the reduced dataset. The model included a spline with seven degrees of freedom. Bold denotes statistical support (95% credible interval (CI) not including zero). Fixed effects were scaled to a mean of zero and standard deviation of one. The model included mother identity and year as random factors.

Variable	Estimate	95% CI
Temperatures at nestling		
Mean Temp. 1	0.141	[-0.201, 0.515]
Mean Temp. 2	0.414	[-0.014, 0.861]
Mean Temp. 3	0.105	[-0.285, 0.500]
Mean Temp. 4	0.216	[-0.195, 0.628]
Mean Temp. 5	-0.174	[-0.556, 0.187]
Mean Temp. 6	0.311	[-0.559, 1.183]
Mean Temp. 7	-0.201	[-0.801, 0.398]
**Laying date**	**-0.302**	**[-0.362, -0.241]**
**Num. Neighbours**	**0.091**	**[0.063, 0.121]**

Table S38: Results from the model analysing the association between number of fledglings and temperature during the fledging period using the reduced dataset. The model included a spline with seven degrees of freedom. Bold denotes statistical support (95% credible interval (CI) not including zero). Fixed effects were scaled to a mean of zero and standard deviation of one. The model included mother identity and year as random factors.

Variable	Estimate	95% CI
Temperatures at fledgling		
**Mean Temp. 1**	**0.633**	**[0.393, 0.885]**
**Mean Temp. 2**	**0.675**	**[0.402, 0.959]**
**Mean Temp. 3**	**0.672**	**[0.413, 0.951]**
**Mean Temp. 4**	**0.679**	**[0.412, 0.964]**
**Mean Temp. 5**	**0.431**	**[0.274, 0.595]**
**Mean Temp. 6**	**1.388**	**[0.846, 1.997]**
**Mean Temp. 7**	**0.249**	**[0.062, 0.450]**
**Laying date**	**-0.011**	**[-0.013, -0.009]**
**Num. Neighbours**	**0.012**	**[0.007, 0.018]**

Table S39: Results from the model analysing the association between fledging success and temperature during the fledgling period using the reduced dataset. The model included a spline with seven degrees of freedom. Bold denotes statistical support (95% credible interval (CI) not including zero). Fixed effects were scaled to a mean of zero and standard deviation of one. The model included mother identity and year as random factors.

Variable	Estimate	95% CI
Temperatures at fledgling		
**Mean Temp. 1**	**2.066**	**[1.434, 2.704]**
**Mean Temp. 2**	**2.750**	**[2.038, 3.454]**
**Mean Temp. 3**	**2.068**	**[1.382, 2.764]**
**Mean Temp. 4**	**2.227**	**[1.515, 2.934]**
**Mean Temp. 5**	**1.152**	**[0.720, 1.603]**
**Mean Temp. 6**	**4.413**	**[2.935, 5.853]**
Mean Temp. 7	0.049	[-0.612, 0.709]
**Laying date**	**-0.194**	**[-0.256, -0.132]**
**Clutch size**	**-0.125**	**[-0.167, -0.083]**
Num. Neighbours	0.013	[-0.025, 0.049]

Table S40: Results from the model analysing the association between number of recruits and temperature during the fledging period using the reduced dataset. The model included a spline with seven degrees of freedom. Bold denotes statistical support (95% credible interval (CI) not including zero). Fixed effects were scaled to a mean of zero and standard deviation of one. The model included mother identity and year as random factors.

Variable	Estimate	95% CI
Temperatures at fledging		
**Mean Temp. 1**	**0.989**	**[0.167, 1.884]**
**Mean Temp. 2**	**1.312**	**[0.381, 2.280]**
**Mean Temp. 3**	**0.998**	**[0.100, 1.893]**
**Mean Temp. 4**	**1.104**	**[0.202, 2.039]**
**Mean Temp. 5**	**0.595**	**[0.037, 1.134]**
**Mean Temp. 6**	**2.567**	**[0.689, 4.544]**
Mean Temp. 7	0.302	[-0.334, 0.933]
**Laying date**	**-0.329**	**[-0.384, -0.277]**
**Num. Neighbours**	**0.090**	**[0.062, 0.120]**

Table S41: Results from the linear models exploring the temporal trends of temperature around half-fall using different size windows.

Variable	Estimate	Std. Error	z	P
		±7 days around half-fall		
Mean Temp.	-0.003	0.011	$F_{1,43}$=0.119	0.731
		±15 days around half-fall		
Mean Temp.	-0.002	0.008	$F_{1,43}$=0.060	0.807
		±20 days around half-fall		
Mean Temp.	-0.003	0.006	$F_{1,43}$=0.264	0.610

Table S42: Results from the linear models analysing the temporal trends of the hypothetical temperatures in the five breeding periods. Bold denotes statistical significance.

**Reproductive Period**	**Estimate**	**SE**	***F***	***P***
		Egg-laying (n=59)		
Year	**0.022**	**0.007**	**$F_{1,57}$=8.695**	**0.004**
		Incubation (n=59)		
Year	**0.023**	**0.007**	**$F_{1,57}$=10.325**	**0.002**
		Hatching period (n=59)		
Year	**0.022**	**0.007**	**$F_{1,57}$=8.004**	**0.006**
		Nestling period (n=59)		
Year	**0.020**	**0.008**	**$F_{1,57}$=5.839**	**0.018**
		Fledging period (n=59)		
Year	**0.019**	**0.009**	**$F_{1,57}$=4.740**	**0.033**

Table S43: Results from the linear mixed models linking the within-individual component of temperature with breeding attempt (i.e. breeding experience). Bold denotes statistical significance.

Variable	Estimate	Std. Error	F	p
		Egg-laying (n=12315)		
Breeding attempt	-0.013	0.010	$F_{1, 12269}$=1.533	0.216
**Age_{juv}**	**0.173**	**0.030**	**$F_{1, 12292}$=33.221**	**<0.001**
		Incubation (n=11643)		
Breeding attempt	**-0.019**	**0.007**	**$F_{1, 11590}$=7.726**	**0.005**
**Age_{juv}**	**0.043**	**0.019**	**$F_{1,11604}$=4.832**	**0.028**
		Hatching (n=11643)		
Breeding attempt	0.008	0.009	$F_{1, 11594}$=0.707	0.400
**Age_{juv}**	**0.173**	**0.027**	**$F_{1, 11611}$=39.848**	**<0.001**
		Nestling (n=11643)		
Breeding attempt	**0.035**	**0.009**	**$F_{1, 11592}$=13.11**	**<0.001**
**Age_{juv}**	**0.239**	**0.027**	**$F_{1, 11608}$=77.45**	**<0.001**
		Fledgling (n=11643)		
Breeding attempt	0.006	0.009	$F_{1, 11591}$=0.498	0.484
**Age_{juv}**	**0.091**	**0.025**	**$F_{1, 11606}$=12.77**	**<0.001**

Table S44: Temperature values that maximise reproductive success obtained by fitting generalised linear mixed models with cubic natural splines with different flexibilities in a Bayesian framework. 95% credible intervals (CI) are shown between brackets. Splines (df=7) are the results shown in the main text. The last column of the table represents the optimal temperatures obtained using the dataset reduced to the years with half-fall date data.

Period	Splines (df=7)	Splines (df=5)	Splines (df=9)	Splines (df=7) Red. dataset
Clutch size				
Egg-laying	11.82 [11.23, 12.58]	11.85 [11.22, 12.40]	11.69 [10.81, 12.70]	11.70 [10.42, 12.60]
Number of fledglings				
Egg-laying	11.61 [10.39, 12.19]	11.55 [11.18, 11.89]	11.22 [10.70, 12.26]	11.05 [10.27, 12.22]
Incubation	16.38 [16.38, 16.38]	16.38 [16.38, 16.38]	16.38 [16.38, 16.38]	15.72 [15.72, 15.72]
Hatching	12.98 [6.45, 19.01]	12.92 [11.00, 19.01]	12.75 [6.45, 19.01]	13.47 [10.50, 17.38]
Nestling	19.10 [19.10, 19.10]	19.10 [19.10, 19.10]	19.10 [19.10, 19.10]	19.10 [19.10, 19.10]
Fledgling	15.79 [13.12, 17.88]	15.80 [14.39, 19.50]	15.71 [13.72, 17.35]	16.62 [12.00, 19.50]
Fledging success				
Egg-laying	10.01 [9.59, 10.46]	10.43 [10.26, 10.58]	10.49 [9.54, 10.81]	10.63 [10.50, 10.76]
Incubation	11.47 [8.78, 16.38]	12.81 [9.28, 16.38]	15.32 [8.84, 16.38]	13.28 [8.71, 15.72]
Hatching	10.65 [10.35, 10.94]	10.90 [10.14, 10.20]	10.74 [10.18, 12.33]	10.81 [10.37, 12.56]
Nestling	19.10 [19.10, 19.10]	7.14 [7.14, 7.14]	19.10 [19.10, 19.10]	19.10 [19.10, 19.10]
Fledgling	13.21 [13.08, 13.30]	13.04 [12.52, 13.24]	13.22 [13.20, 13.25]	13.18 [13.12, 13.21]
Number of recruits				
Egg-laying	9.60 [7.95, 11.60]	9.34 [7.86, 11.40]	9.61 [7.96, 11.63]	9.75 [8.55, 11.36]
Incubation	11.43 [8.17, 13.40]	11.17 [6.83, 13.40]	11.59 [8.03, 13.60]	12.34 [8.20, 13.90]
Hatching	13.02 [6.47, 19.01]	12.14 [6.47, 19.01]	12.20 [6.45, 19.01]	11.23 [10.76, 12.60]
Nestling	19.10 [19.10, 19.10]	19.10 [19.10, 19.10]	19.10 [19.10, 19.10]	19.10 [19.10, 19.10]
Fledgling	13.53 [10.98, 19.5]	13.35 [8.69, 19.5]	12.66 [8.69, 17.28]	14.61 [11.34, 19.5]

Table S45: Temperatures at egg-laying that maximise reproductive success using three complementary approaches. Values in brackets represent the 95% credible interval obtained from the posterior distribution of a Bayesian model.

Model	Clutch size	Num. fledglings	Fledgling success	Num. Recruits
Bayesian w. year as random	11.82 [11.23, 12.58]	11.61 [10.39, 12.19]	10.01 [9.59, 10.46]	9.60 [7.95, 11.60]
Random slopes and intercepts freq.	11.32	10.41	9.90	9.88
Bayesian year-centred	11.11 [9.50, 11.58]	10.84 [9.53, 11.31]	9.80 [9.56, 10.18]	8.70 [3.81, 17.77]

Table S46: Temperatures during egg-laying that minimise mismatch with the caterpillar peak obtained from three complementary modelling approaches: a regular mixed effect model with splines, a random slope model quadratic terms and a mixed effect model using the year centred values of mismatch. Values between brackets represent the credible intervals from the posterior distribution of a Bayesian model. The random slope model was fitted in a frequentist framework.

Model	Laying	Incubation	Hatching	Nestling
Bayesian w. year as random	10.82 [10.72, 10.94]	13.67 [13.43, 13.96]	14.01 [13.87, 14.14]	13.95 [12.30, 13.96]
Random slopes and intercepts freq.	10.18	12.27	15.12	17.49
Bayesian year-centred	10.94 [10.75, 11.11]	13.68 [13.45, 13.97]	13.94 [13.76, 14.09]	14.78 [12.30, 19.10]

REFERENCES

1. C. M. Herrera, A. Núñez, J. Valverde, C. Alonso, Body mass decline in a Mediterranean community of solitary bees supports the size shrinking effect of climatic warming. *Ecology* **104**, e4128 (2023).

2. D. López-Idiáquez, C. Teplitsky, A. Grégoire, A. Fargevieille, M. Del Rey, C. De Franceschi, A. Charmantier, C. Doutrelant, Long-term decrease in coloration: A consequence of climate change? *Am. Nat.* **200**, 32–47 (2022).

3. E. F. Cole, C. E. Regan, B. C. Sheldon, Spatial variation in avian phenological response to climate change linked to tree health. *Nat. Clim. Chang.* **11**, 872–878 (2021).

4. J. A. Sheridan, D. Bickford, Shrinking body size as an ecological response to climate change. *Nat. Clim. Chang.* **1**, 401–406 (2011).

5. V. Radchuk, T. Reed, C. Teplitsky, M. van de Pol, A. Charmantier, C. Hassall, P. Adamík, F. Adriaensen, M. P. Ahola, P. Arcese, J. M. Avilés, J. Balbontín, K. S. Berg, A. Borras, S. Burthe, J. Clobert, N. Dehnhard, F. de Lope, A. A. Dhondt, N. J. Dingemanse, H. Doi, T. Eeva, J. Fickel, I. Filella, F. Fossøy, A. E. Goodenough, S. J. G. Hall, B. Hansson, M. Harris, D. Hasselquist, T. Hickler, J. Joshi, H. Kharouba, J. G. Martínez, J.-B. Mihoub, J. A. Mills, M. Molina-Morales, A. Moksnes, A. Ozgul, D. Parejo, P. Pilard, M. Poisbleau, F. Rousset, M.-O. Rödel, D. Scott, J. C. Senar, C. Stefanescu, B. G. Stokke, T. Kusano, M. Tarka, C. E. Tarwater, K. Thonicke, J. Thorley, A. Wilting, P. Tryjanowski, J. Merilä, B. C. Sheldon, A. P. Møller, E. Matthysen, F. Janzen, F. S. Dobson, M. E. Visser, S. R. Beissinger, A. Courtiol, S. Kramer-Schadt, Adaptive responses of animals to climate change are most likely insufficient. *Nat. Commun.* **10**, 1–14 (2019).

6. IPCC, 2023: Summary for Policymakers, in Climate Change 2023: Synthesis Report. Contribution of Working Groups I, II and III tothe Sixth Assessment Report of the Intergovernmental Panel on Climate Change, Core Writing Team, H. Lee, J. Romero Eds. (IPCC, Geneva, Switzerland, 2023), pp. 1–34.

7. M. E. Visser, L. J. M. Holleman, P. Gienapp, Shifts in caterpillar biomass phenology due to climate change and its impact on the breeding biology of an insectivorous bird. *Oecologia* **147**, 164–172 (2006).

8. A. Charmantier, R. H. McCleery, L. R. Cole, C. M. Perrins, L. E. B. Kruuk, B. C. Sheldon, Adaptive phenotypic plasticity in response to climate change in a wild bird population. *Science* **320**, 800–803 (2008).

9. C. Both, M. Van Asch, R. G. Bijlsma, A. B. Van Den Burg, M. E. Visser, Climate change and unequal phenological changes across four trophic levels: Constraints or adaptations? *J. Anim. Ecol.* **78**, 73–83 (2009).

10. C. Youngflesh, G. A. Montgomery, J. F. Saracco, D. A. W. Miller, R. P. Guralnick, A. H. Hurlbert, R. B. Siegel, R. LaFrance, M. W. Tingley, Demographic consequences of phenological asynchrony for North American songbirds. *Proc. Natl. Acad. Sci. U.S.A.* **120**, e2221961120 (2023).

11. R. Hickling, D. B. Roy, J. K. Hill, R. Fox, C. D. Thomas, The distributions of a wide range of taxonomic groups are expanding polewards. *Glob. Chang. Biol.* **12**, 450–455 (2006).

12. M. W. Tingley, W. B. Monahan, S. R. Beissinger, C. Moritz, Birds track their Grinnellian niche through a century of climate change. *Proc. Natl. Acad. Sci. U.S.A.* **106**, 19637–19643 (2009).

13. V. Devictor, C. Van Swaay, T. Brereton, L. Brotons, D. Chamberlain, J. Heliölä, S. Herrando, R. Julliard, M. Kuussaari, Å. Lindström, J. Reif, D. B. Roy, O. Schweiger, J. Settele, C. Stefanescu, A. Van Strien, C. Van Turnhout, Z. Vermouzek, M. WallisDeVries, I. Wynhoff, F. Jiguet, Differences in the climatic debts of birds and butterflies at a continental scale. *Nat. Clim. Chang.* **2**, 121–124 (2012).

14. M. L. Pinsky, B. Worm, M. J. Fogarty, J. L. Sarmiento, S. A. Levin, Marine taxa track local climate velocities. *Science* **341**, 1239–1242 (2013).

15. T. Amano, R. P. Freckleton, S. A. Queenborough, S. W. Doxford, R. J. Smithers, T. H. Sparks, W. J. Sutherland, Links between plant species' spatial and temporal responses to a warming climate. *Proc. R. Soc. B* **281**, 20133017 (2014).

16. J. B. Socolar, P. N. Epanchin, S. R. Beissinger, M. W. Tingley, Phenological shifts conserve thermal niches in North American birds and reshape expectations for climate-driven range shifts. *Proc. Natl. Acad. Sci. U.S.A.* **114**, 12976–12981 (2017).

17. M. H. C. Neate-Clegg, B. A. Tonelli, M. W. Tingley, Advances in breeding phenology outpace latitudinal and elevational shifts for North American birds tracking temperature. *Nat. Ecol. Evol.* **8**, 2027–2036 (2024).

18. B. C. Sheldon, L. E. B. Kruuk, S. C. Alberts, The expanding value of long-term studies of individuals in the wild. *Nat. Ecol. Evol.* **6**, 1799–1801 (2022).

19. L. D. Bailey, M. Van De Pol, F. Adriaensen, A. Arct, E. Barba, P. E. Bellamy, S. Bonamour, J.-C. Bouvier, M. D. Burgess, A. Charmantier, C. Cusimano, B. Doligez, S. M. Drobniak, A. Dubiec, M. Eens, T. Eeva, P. N. Ferns, A. E. Goodenough, I. R. Hartley, S. A. Hinsley, E. Ivankina, R. Juškaitis, B. Kempenaers, A. B. Kerimov, C. Lavigne, A. Leivits, M. C. Mainwaring, E. Matthysen, J.-Å. Nilsson, M. Orell, S. Rytkönen, J. C. Senar, B. C. Sheldon, A. Sorace, M. J. Stenning, J. Török, K. Van Oers, E. Vatka, S. J. G. Vriend, M. E. Visser, Bird populations most exposed to climate change are less sensitive to climatic variation. *Nat. Commun.* **13**, 2112 (2022).

20. C. E. Regan, B. C. Sheldon, Phenotypic plasticity increases exposure to extreme climatic events that reduce individual fitness. *Glob. Chang. Biol.* **29**, 2968–2980 (2023).

21. A. J. van Noordwijk, R. H. McCleery, C. M. Perrins, Selection for the timing of great tit breeding in relation to caterpillar growth and temperature. *J. Anim. Ecol.* **64**, 451 (1995).

22. P. Marrot, D. Garant, A. Charmantier, Multiple extreme climatic events strengthen selection for earlier breeding in a wild passerine. *Philos. Trans. R. Soc. B* **372**, 20160372 (2017).

23. T. E. Reed, V. Grøtan, S. Jenouvrier, B.-E. Sæther, M. E. Visser, Population growth in a wild bird is buffered against phenological mismatch. *Science* **340**, 488–491 (2013).

24. C. M. Perrins, Tits and their caterpillar food supply. *Ibis* **133**, 49–54 (1991).

25. T. A. Wilkin, L. E. King, B. C. Sheldon, Habitat quality, nestling diet, and provisioning behaviour in great tits *Parus major*. *J. Avian Biol.* **40**, 135–145 (2009).

26. C. Sinkovics, G. Seress, I. Pipoly, E. Vincze, A. Liker, Great tits feed their nestlings with more but smaller prey items and fewer caterpillars in cities than in forests. *Sci. Rep.* **11**, 24161 (2021).

27. S. Rytkönen, E. J. Vesterinen, C. Westerduin, T. Leviäkangas, E. Vatka, M. Mutanen, P. Välimäki, M. Hukkanen, M. Suokas, M. Orell, From feces to data: A metabarcoding method for analyzing consumed and available prey in a bird-insect food web. *Ecol. Evol.* **9**, 631–639 (2019).

28. J. D. Shutt, J. A. Nicholls, U. H. Trivedi, M. D. Burgess, G. N. Stone, J. D. Hadfield, A. B. Phillimore, Gradients in richness and turnover of a forest passerine's diet prior to breeding: A mixed model approach applied to faecal metabarcoding data. *Mol. Ecol.* **29**, 1199–1213 (2020).

29. L. C. Evans, M. D. Burgess, S. G. Potts, W. E. Kunin, T. H. Oliver, Population links between an insectivorous bird and moths disentangled through national-scale monitoring data. *Ecol. Lett.* **27**, e14362 (2024).

30. C. Jarrett, L. L. Powell, H. McDevitt, B. Helm, A. J. Welch, Bitter fruits of hard labour: Diet metabarcoding and telemetry reveal that urban songbirds travel further for lower-quality food. *Oecologia* **193**, 377–388 (2020).

31. A. E. Hinks, E. F. Cole, K. J. Daniels, T. A. Wilkin, S. Nakagawa, B. C. Sheldon, Scale-dependent phenological synchrony between songbirds and their caterpillar food source. *Am. Nat.* **186**, 84–97 (2015).

32. L. M. Morley, S. Crofts, E. F. Cole, B. C. Sheldon, Quantifying phenology in the deciduous tree and phytophagous insect system: A methodological comparison. *Ecol. Evol.* **15**, e71821 (2025).

33. S. J. Thackeray, P. A. Henrys, D. Hemming, J. R. Bell, M. S. Botham, S. Burthe, P. Helaouet, D. G. Johns, I. D. Jones, D. I. Leech, E. B. Mackay, D. Massimino, S. Atkinson, P. J. Bacon, T. M. Brereton, L. Carvalho, T. H. Clutton-Brock, C. Duck, M. Edwards, J. M. Elliott, S. J. G. Hall, R. Harrington, J. W. Pearce-Higgins, T. T. Høye, L. E. B. Kruuk, J. M. Pemberton, T. H. Sparks, P. M. Thompson, I. White, I. J. Winfield, S. Wanless, Phenological sensitivity to climate across taxa and trophic levels. *Nature* **535**, 241–245 (2016).

34. S. J. G. Vriend, V. Grøtan, M. Gamelon, F. Adriaensen, M. P. Ahola, E. Álvarez, L. D. Bailey, E. Barba, J. Bouvier, M. D. Burgess, A. Bushuev, C. Camacho, D. Canal, A. Charmantier, E. F. Cole, C. Cusimano, B. F. Doligez, S. M. Drobniak, A. Dubiec, M. Eens, T. Eeva, K. E. Erikstad, P. N. Ferns, A. E. Goodenough, I. R. Hartley, S. A. Hinsley, E. Ivankina, R. Juškaitis, B. Kempenaers, A. B. Kerimov, J. A. Kålås, C. Lavigne, A. Leivits, M. C. Mainwaring, J. Martínez-Padilla, E. Matthysen, K. Van Oers, M. Orell, R. Pinxten, T. K. Reiertsen, S. Rytkönen, J. C. Senar, B. C. Sheldon, A. Sorace, J. Török, E. Vatka, M. E. Visser, B. Sæther, Temperature synchronizes temporal variation in laying dates across European hole-nesting passerines. *Ecology* **104**, e3908 (2023).

35. P. de Villemereuil, A. Charmantier, D. Arlt, P. Bize, P. Brekke, L. Brouwer, A. Cockburn, S. D. Côté, F. S. Dobson, S. R. Evans, M. Festa-Bianchet, M. Gamelon, S. Hamel, J. Hegelbach, K. Jerstad, B. Kempenaers, L. E. B. Kruuk, J. Kumpula, T. Kvalnes, A. G. McAdam, S. E. McFarlane, M. B. Morrissey, T. Pärt, J. M. Pemberton, A. Qvarnström, O. W. Røstad, J. Schroeder, J. C. Senar, B. C. Sheldon, M. Van De Pol, M. E. Visser, N. T. Wheelwright, J. Tufto, L.-M. Chevin, Fluctuating optimum and temporally variable selection on breeding date in birds and mammals. *Proc. Natl. Acad. Sci. U.S.A.* **117**, 31969–31978 (2020).

36. K. Tona, K. Voemesse, O. N'nanlé, O. E. Oke, Y. A. E. Kouame, A. Bilalissi, H. Meteyake, O. M. Oso, Chicken incubation conditions: Role in embryo development, physiology and adaptation to the post-hatch environment. *Front. Physiol.* **13**, 895854 (2022).

37. G. R. Hepp, R. A. Kennamer, M. H. Johnson, Maternal effects in Wood Ducks: Incubation temperature influences incubation period and neonate phenotype. *Funct. Ecol.* **20**, 308–314 (2006).

38. A. J. Clauser, S. B. McRae, Plasticity in incubation behavior and shading by king rails *Rallus elegans* in response to temperature. *J. Avian Biol.* **48**, 479–488 (2017).

39. G. R. Hepp, R. A. Kennamer, Warm is better: Incubation temperature influences apparent survival and recruitment of wood ducks (*Aix sponsa*). *PLOS ONE* **7**, e47777 (2012).

40. A. Nord, J.-Å. Nilsson, Long-term consequences of high incubation temperature in a wild bird population. *Biol. Lett.* **12**, 20160087 (2016).

41. M. E. Visser, A. J. van Noordwijk, J. M. Tinbergen, C. M. Lessells, Warmer springs lead to mistimed reproduction in great tits (Parus major). *Proc. R. Soc. B* **265**, 1867–1870 (1998).

42. W. Cresswell, R. H. McCleery, How great tits maintain synchronization of their hatch date with food supply in response to long-term variability in temperature. *J. Anim. Ecol.* **72**, 356–366 (2003).

43. E. G. Simmonds, B. C. Sheldon, T. Coulson, E. F. Cole, Incubation behavior adjustments, driven by ambient temperature variation, improve synchrony between hatch dates and caterpillar peak in a wild bird population. *Ecol. Evol.* **7**, 9415–9425 (2017).

44. D. W. Thomas, J. Blondel, P. Perret, M. M. Lambrechts, J. R. Speakman, Energetic and fitness costs of mismatching resource supply and demand in seasonally breeding birds. *Science* **291**, 2598–2600 (2001).

45. M. E. Visser, L. J. M. Holleman, S. P. Caro, Temperature has a causal effect on avian timing of reproduction. *Proc. R. Soc. B* **276**, 2323–2331 (2009).

46. S. P. Caro, S. V. Schaper, A. Dawson, P. J. Sharp, P. Gienapp, M. E. Visser, Is microevolution the only emergency exit in a warming world? Temperature influences egg laying but not its underlying mechanisms in great tits. *Gen. Comp. Endocrinol.* **190**, 164–169 (2013).

47. J. Nilsson, H. Källander, Leafing phenology and timing of egg laying in great tits *Parus major* and blue tits *P. caeruleus*. *J. Avian Biol.* **37**, 357–363 (2006).

48. P. Bourgault, D. Thomas, P. Perret, J. Blondel, Spring vegetation phenology is a robust predictor of breeding date across broad landscapes: A multi-site approach using the Corsican blue tit (*Cyanistes caeruleus*). *Oecologia* **162**, 885–892 (2010).

49. E. F. Cole, P. R. Long, P. Zelazowski, M. Szulkin, B. C. Sheldon, Predicting bird phenology from space: Satellite-derived vegetation green-up signal uncovers spatial variation in phenological synchrony between birds and their environment. *Ecol. Evol.* **5**, 5057–5074 (2015).

50. S. V. Schaper, C. Rueda, P. J. Sharp, A. Dawson, M. E. Visser, Spring phenology does not affect timing of reproduction in the great tit (*Parus major*). *J. Exp. Biol.* **214**, 3664–3671 (2011).

51. E. Matthysen, F. Adriaensen, P. Van De Kerckhove, K. Vandekerkhove, Great and blue tit laying dates vary with fine-scale variation in local tree composition but not tree budburst. *J. Ornithol.* **162**, 709–722 (2021).

52. N. E. van Dis, G.-J. Sieperda, V. Bansal, B. van Lith, B. Wertheim, M. E. Visser, Phenological mismatch affects individual fitness and population growth in the winter moth. *Proc. R. Soc. B* **290**, 20230414 (2023).

53. C. M. Perrins, R. H. McCleery, Laying dates and clutch size in the great tit. *Wilson Bull.* **101**, 236–253 (1989).

54. S. Bouwhuis, B. C. Sheldon, S. Verhulst, A. Charmantier, Great tits growing old: Selective disappearance and the partitioning of senescence to stages within the breeding cycle. *Proc. R. Soc. B* **276**, 2769–2777 (2009).

55. R Core Team, R: A Language and Environment for Statistical Computing (R Foundation for Statistical Computing, 2023).

56. D. Bates, M. Maechler, B. M. Bolker, lme4: Linear mixed-effects models using S4 classes, R package version 0.999999-2 (2013); www.scirp.org/reference/referencespapers?referenceid=1242679.

57. P. C. Bürkner, brms: An R package for Bayesian multilevel models using stan. *J. Stat. Softw.* **80**, 1–28 (2017).

58. D. Lüdecke, ggeffects: Tidy data frames of marginal effects from regression models. *J. Open SOurce Softw.* **3**, 772 (2018).

59. M. Mayer, confintr: Confidence Intervals (2023); https://mayer79.r-universe.dev/confintr.

60. L. D. Bailey, M. Van De Pol, climwin: An R toolbox for climate window analysis. *PLOS ONE* **11**, e0167980 (2016).

61. E. G. Simmonds, E. F. Cole, B. C. Sheldon, Cue identification in phenology: A case study of the predictive performance of current statistical tools. *J. Anim. Ecol.* **88**, 1428–1440 (2019).

62. M. van de Pol, J. Wrigth, A simple method for distinguishing within- versus between-subject effects using mixed models. *Anim. Behav.* **77**, 753–758 (2009).
